# Waves of novelties in the expansion into the adjacent possible

**DOI:** 10.1371/journal.pone.0179303

**Published:** 2017-06-08

**Authors:** Bernardo Monechi, Ãlvaro Ruiz-Serrano, Francesca Tria, Vittorio Loreto

**Affiliations:** 1 ISI Foundation, Via Alassio 11C, 10126 Torino, Italy; 2 Sapienza University of Rome, Physics Dept., Piazzale Aldo Moro 5, 00185 Roma, Italy; University of Sydney, AUSTRALIA

## Abstract

The emergence of novelties and their rise and fall in popularity is an ubiquitous phenomenon in human activities. The coexistence of popular evergreens with novel and sometimes ephemeral trends pervades technological, scientific and artistic production. Though this phenomenon is very intuitively captured by our common sense, a comprehensive explanation of how waves of novelties are not hampered by well established old-comers is still lacking. Here we first quantify this phenomenology by empirically looking at different systems that display innovation at very different levels: the creation of hashtags in Twitter, the evolution of online code repositories, the creation of texts and the listening of songs on online platforms. In all these systems surprisingly similar patterns emerge as the non-trivial outcome of two contrasting forces: the tendency of retracing already explored avenues (exploit) and the inclination to explore new possibilities. These findings are naturally explained in the framework of the expansion of the adjacent possible, a recently introduced theoretical framework that postulates the restructuring of the space of possibilities conditional to the occurrence of innovations. The predictions of our theoretical framework are borne out in all the phenomenologies investigated, paving the way to a better understanding and control of innovation processes.

## Introduction

Every field of human activities is characterized by the emergence of novelties. The introduction of a new idea expands the possibility to explore newer concepts and, once introduced, the new idea has to compete with other concomitant ideas. Only some of them will stand out from the crowd but those will have higher chances to become even more successful through a mechanism of reinforcement, often denoted rich-get-richer: [1–3] the chances of increasing the popularity of a given element of a system grow with its previous popularity. If this is the fate of very few ideas, the vast majority of new ideas never become known enough within the population, destined, as they are, to fade into oblivion. In the struggle for survival, junior ideas often have to compete with older and more popular items that have already acquired a recognised status, the so-called evergreens, more likely focusing the attention of the population. Though the rich-get-richer mechanism would predict that junior ideas would never become popular, still one very frequently witnesses the birth and emergence of waves of novelties: a new hit, a new book, a new technology, a new social structure, etc. And sometimes novelties could become even more popular than their predecessors. In the artistic domain, for instance, popular music is somehow paradigmatic. Milestones are still listened and appreciated by many people, while on the other hand new genres and artists can rapidly become worldwide famous gathering the attention of the vast majority of the public. This example is obviously not isolated and many other insightful examples can be found, from the emergence of new fundamental works in the scientific production to the paradigm shifts in art movements.

The study of innovation processes has been extensively tackled by the scientific community [4–10], with a focus, in relatively recent times, to the study of the dynamics of popularity. Many efforts have been devoted to the introduction of suitable metrics to predict the future popularity of the elements of various systems from early conditions [11–17] or to identify the features of innovative or creative elements [18, 19]. From the modelling perspective, the attention has been focused in the prediction of the future importance of an element by fitting the early stages of its dynamics [20–22] or in the characterization of the microscopic dynamical patterns of single elements [23–26]. To the best of our knowledge, not much work focused so far on the competition between new and old elements and on how the global popularity distribution is shaped by this process. In [27, 28] it has been shown how the interplay between this competition and the limited amount of elements that can be reminded by an individual can explain both the lifetime and the popularity distribution of Internet memes. In [29] a stylized model for these exploration-exploitation situations has been proposed that reveals the existence of an optimal regime. More recently, in [30] it has been shown how, in an economic framework, the competition between the exploitation of existing goods and the introduction of innovations can shape the topology of the products’ network.

The usual metaphor *Exploit-Explore* nicely applies here. Is it better to stay in a given situation (*exploit* a given technology, keeping a given job, constantly retracing old steps, stick to given paradigms, etc.) or move away and *explore* new avenues (invest in a new technology, accept a radically new job, constantly looking for new experiences, challenge current paradigms, etc.)? Here intuition suggests that both extremes are dangerous and presumably innovation cannot take place in any of those *pure* positions. A hybrid strategy instead, which wisely blends those two opposite attitudes, seems to be obviously in order. But how? What is the right balance point? And even more importantly, can we observe this balance in action in real life? Addressing these questions is the main aim of this paper.

With these questions in mind, here we focus on the competition between *recent* and *old* elements, and investigate the dynamics leading to the emergence of new trending elements capable of achieving popularity, though for a limited time. To this end we introduce several metrics aiming at identifying the bias towards young or less recent elements in the distribution of popularity, either long-lasting (global time scale) or ephemeral (local time-frames). We test then in five different contexts mirroring human activities. We focus in particular on the following datasets. The Last.Fm Music Recommendation dataset [31], in which a novelty occurs at the individual scale as the listening of a new song by a given user, the Wikipedia Article Texts dataset [32] and the Gutenberg English Corpus [33], in which innovations are represented by words appearing for the first time in the texts but not necessary in the world (typically the word already exists in the English dictionary). On the other hand Github [34] and Twitter [16] provide datasets in which new elements (new code repositories in Github and new hashtags in Twitter) can be created from scratch and contribute to the growth of the shared set of resources users can eventually adopt. All the datasets used in this work are freely available for download at [35] or at [36].

Our findings reveal that in all the datasets considered old and popular elements are not always the most successful. Recently introduced elements may always become more popular than older ones in short time-frames, effectively creating waves of popularity. What emerges is a quite complex pattern where public attention is captured at different points in time by either famous and old elements, or by recent and pretty unknown novelties or also by elements lying somewhere in between these two cases. This oscillation in popularity between old and new elements is observed in all the datasets we considered, with strikingly similar regularities.

In order to unfold the above mentioned phenomenology we exploit a new framework based on the notion of *Adjacent Possible*. Theorised by Kauffman to explain molecular and biological evolution [37], and extended afterwards to other contexts, the adjacent possible consists of all things that are one step away from what already exists. The interesting property of this space of possibilities is that it is dynamical and it gets restructured every time a novelty appears. In other words, the space of possibilities expands whenever its elements are taken from it into the real world in a constant dynamical interplay between the *actual* and the *possible* [38]. In [39] the dynamics of the adjacent possible has been modelled extending the Polya’s Urn [40, 41] framework, and it was proved to be successful in explaining key empirical observations like the existence of correlated innovation and their clustering in time. Here we show that a simple generalisation of the original model turns out to be able to predict the emergence and evolution of waves of novelties. From the comparison of those predictions with the empirical data mentioned above we conclude that real systems lie in between the drive to *explore* the adjacent possible and the need to *exploit* the already known.

## Results

In [39] it was shown that large databases witnessing human activities share many statistical features describing the emergence of novelties and innovations. We here push further this analysis, by focusing on additional measures more directly related to the emergence and success of innovations or novelties, and considering additional datasets witnessing the generation and recombination of new resources. All together, we here consider the following datasets, each consisting in a sequence of time-ordered elements, created or adopted by the respective community of users (detailed information about the various datasets can be found in Section A of S1 File): the *Last.fm Music Tracks* dataset, in which the elements are the songs of the online platform “Last.fm”, ordered according to the time each track has been listened by a user; the *Twitter Hashtags* dataset, originally collected in [16], whose elements are posts containing at least a hashtag ordered by posting time; the *GitHub Archive* dataset, whose elements are GitHub repositories, ordered by the time a GitHub user has created or operated on a code repository; the *Wikipedia Article Text* dataset, in which elements are words, and where a small subsample of Wikipedia articles, joint together in order to create a unique long text, is considered; the *Gutenberg Corpus* data, in which elements are again words and we joined together all the books in a unique corpus. Note that in Wikipedia we were able to correctly attach a time stamp to each article according to the last edit recorded, so that the sequence of words of the unique text resembles the true time-ordered sequence of words written by Wikipedia editors. This is not the case for the Gutenberg Corpus whose books lack a well-defined time stamp. In this case the whole sequence is obtained by joining books in a random order. We remark that the lack of a well-defined time stamp for each book within the Gutenberg Corpus does not imply that the order of words in the individual texts is random. The correlations at the level of the individual text are well preserved and for the sake of our analysis we do not expect major changes in our results if we considered the correct time-ordering of the different texts within the corpus. The reason for this is that we do not expect major correlations in place across different texts (for instance the adoption of a neologism in a specific novel could have triggered the adoption of the new word in the subsequent novels). We consequently do not expect a statistically significant effect on our results. This hypothesis can be confirmed by looking at the equivalent case of Wikipedia (see Section D of S1 File). Since hardly any difference is noticed between the two cases we can safely conclude that the phenomenology observed in the Gutenberg dataset is genuine and do not crucially depend on the way we constructed the time ordering of texts within the Gutenberg corpus (see also [39, 42]).

Before proceeding further a word of caution is in order. Some of the considered systems, e.g., Last.fm, have implemented recommendation algorithms that suggest users specific tags to be adopted (e.g., similar tracks of users’ top songs, popular tracks similar to users’ top songs, top tracks of users’ neighbours, etc.) This recommendation procedures might in principle bias the exploration/exploitation interplay. For instance a recommendation engine could suggest users to adopt already popular elements, hence strengthening the reinforcement mechanism and slowing down the growth of the number of the distinct elements *D*(*t*). Similarly, the recommendation could highlight elements related to what a particular user already knows and adopts, hence increasing the semantic correlations between elements. Though the detailed functioning of these recommendation engines is not known in details, a detailed analysis of these effects would have a value *per se*. We expect anyway that these recommendation procedures would not drastically change our main conclusions, affecting for instance only the numerical values of the model parameters, not the universal mechanisms our modelling scheme is capturing.

Let us now get back to the exposition of the results. A first statistical signature of systems where innovation is present is the Heap’s Law [43], i.e., a sub-linear growth of the total number of different elements as a function of the total number of elements in the sequence of events: by indicating with *S*(*t*) the sequence of the elements in a given dataset at time *t* (where the time in counted as the length of the sequence), and by *D*(*t*) the total number of distinct elements in *S*(*t*), *D*(*t*) = *t*^*β*^ with *β* < 1. A second related statistical property is the Zipf’s Law for the Frequency-Rank distribution. The tail of this distribution can be approximated as a power-law, *f*(*R*)∼*R*^−*α*^, with the exponent related to the one characterising the Heaps’ law, namely *α* = 1/*β* [44, 45]. These two statistical signatures, as well as their relationships, are naturally observed in all the datasets we considered (we refer to Section B of S1 File), with the value of the exponent *β* ranging between 0.5 and 0.8, consistent, for the textual databases, with those usually found for English texts. Another important signature of innovation processes is the presence of *semantic correlations*, i.e., the tendency of semantically related elements to appear clustered in the time sequence. In [39] semantic correlations have been quantified through the distribution of the time intervals between semantically related elements, *f*(*l*), and an entropic measure *S*(*k*), again quantifying the average degree of clustering of semantically related elements that have appeared *k* times in the sequence (we refer to Section B of S1 File for further details).

### Statistical patterns in the emergence of successful novelties

**Fig 1:**
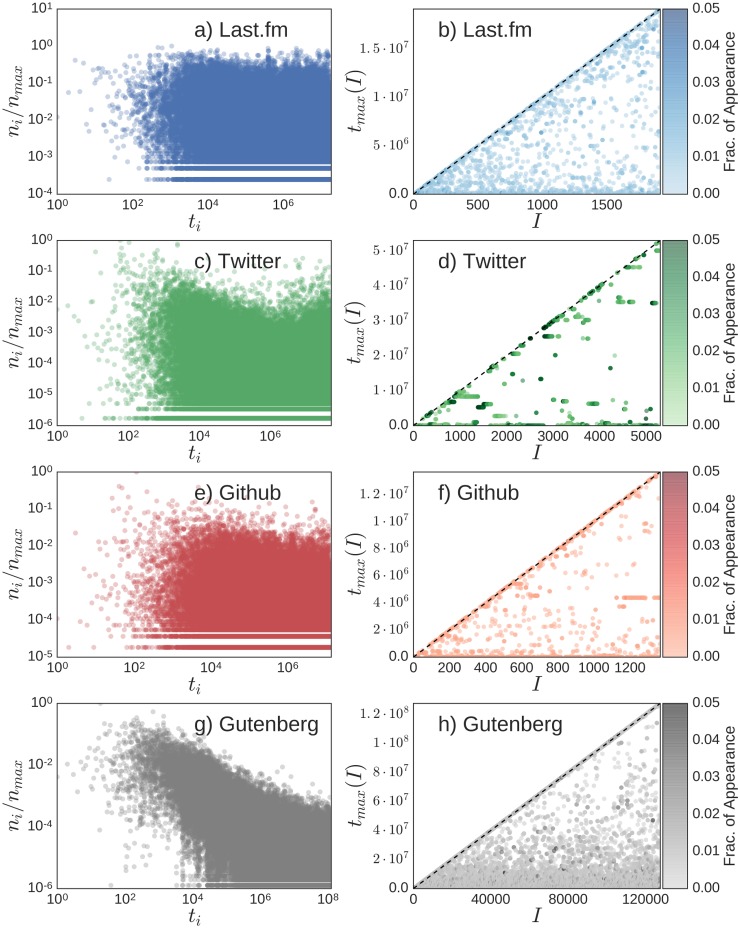
Beyond rich-get-richer. (Left-Panels) Normalised frequency of occurrence *n*_*i*_ of each element in *i* ∈ *S* as a function of its first appearance time *t*_*i*_. (Right-Panels) For each interval of length Δ*τ* we plot of the first appearance time of the most popular element within the interval. Data are shown for the Last.fm (a-b), Twitter (c-d), Github (e-f) and Gutenberg (g-h) datasets. Colour is coding for the fraction of time the successful element has appeared within the corresponding interval. The length of the interval is Δ*τ* = 10000 for non-text datasets and Δ*τ* = 1000 for the Gutenberg one.

Our aim here is that of quantifying waves of popularity of novelties. To this end it is important to relate the success of a given novelty to its first appearance time. Successful waves correspond to relatively young novelties able to capture the attention of many users in a relatively short period of time. Given the sequence *S* of events, we denote with *t*_*i*_ the first appearance time of the *i*-th element of *S*. Fig 1 gives a view of the variability of the popularity of the elements in four of the datasets above (data for Wikipedia are shown in section D of S1 File). In particular the left panels report the number of occurrences *n*_*i*_ of each element of *i* ∈ *S*, normalised through *n*^*max*^ = max_*i*_ *n*_*i*_, as a function of the first appearance times *t*_*i*_. It is evident how also recently introduced element can be extremely successful. In order to get a deeper insight into this phenomenology, we divide the whole history in *T*/Δ*τ* intervals of duration Δ*τ* (*T* being the total length of *S*) and in each interval we identify the most successful element. The right panels report the first appearance time *t*_*i*_ for the most popular element in each time interval *I*. If the most successful elements were always the oldest ones all the points should lay on the *x*-axis. On the other hand if the most recent element was the trendy one all the point should lay on the *t*_*i*_ = *I*Δ*τ* line. It is evident the existence of waves of recent novelties as witnessed by a hybrid situation in between the two extreme cases just defined. We refer to Section D of the S1 File for a detailed discussion of the textual cases (Wikipedia and Gutenberg).

**Fig 2:**
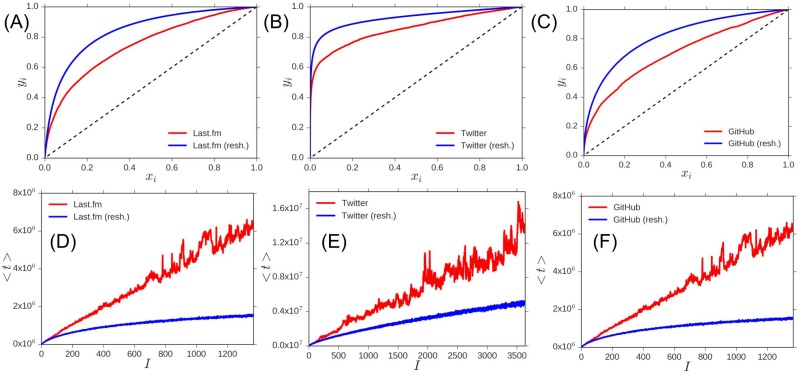
Youth coefficient and Gini-like coefficient. (A-B-C) Curves for the computation of the *Gini-like* coefficient: on the axes, *x*_*i*_ is the relative rank of an element according to its appearance time (older elements on lower ranks), and *y*_*i*_ is the cumulative frequency of occurrence of elements up to *i* in *S*. Results are shown for the Last.fm (A), Twitter (B) and GitHub (C) datasets. (D-E-F) Graphs for computing the *Youth* coefficient: Average introduction time of the elements within an interval of *S* as a function of the interval index, for the Last.fm (D), Twitter (E) and GitHub (F) datasets. In each panel, red curves represents the real datasets while blue curves represent the reshuffling of the data in which *S* has been randomly re-ordered.

We can now quantify the observed phenomenology by introducing several metrics characterising the variability in time of the popularity of the elements in *S* (we refer to the S1 File for further details). Our first metrics is a *Gini-like* coefficient [46]. Originally introduced to quantify inequalities among values of a frequency distribution (for example, levels of income), the Gini index allows us to estimate the deviations from the uniform distribution of success for all the elements in *S*. In our case we define our Gini-like index as follows. Let us consider a two-dimensional area through the two quantities *x*_*i*_, the relative rank of an element according to its appearance time (older elements on lower ranks), and *y*_*i*_, the cumulative frequency of occurrence of elements up to *i* in *S*. In this space we compute the coefficient *G* as the area between the *y*_*i*_(*x*_*i*_) curve and the *y*_*i*_ = *x*_*i*_ line. If the curve *y*_*i*_(*x*_*i*_) > *x*_*i*_ then *G* > 0, indicating that old elements share the largest part of popularity, while if *y*_*i*_(*x*_*i*_) < *x*_*i*_ then *G* < 0, and recent elements are the trendy ones. The (*x*_*i*_,*y*_*i*_) curves for the non-textual data are shown in Fig 2, while these curves for all the datasets are shown in Fig C of S1 File. *G* is bounded in the interval [−1,1]. (We refer to the S1 File for a rigorous definition of *G* and specific examples). Table 1 shows the results of *G* for the above defined datasets and their reshuffled version in which the elements of S are randomly reordered. In all cases the Gini-like coefficient is smaller than the corresponding value for a reshuffled sequence, signalling the occurrence of waves of novelties. The above results are very robust and they can be confirmed also looking at slightly different observables. We introduce in particular the *Youth Coefficient* *Y* and the *Recentness (of the Trending Elements)* *R* as follows (we refer to the S1 File for the rigorous definitions).

**Table 1:** Variability of popularity metrics.

	*G*	*G* (resh.)	*Y*	*Y* (resh.)	*R*	*R* (resh.)	〈*h*〉	〈*h*〉 (resh.)
Last.fm	0.491	0.685	0.379	0.056	0.516	7.46 × 10^−6^	0.982	0.997
Twitter	0.405	0.628	0.463	0.089	0.448	5.24 × 10^−5^	0.961	0.993
GitHub	0.706	0.85	0.339	0.128	0.386	2.34 × 10^−6^	0.907	0.945
Wikipedia	0.889	0.907	0.035	0.022	0.02	1.78 × 10^−6^	0.930	0.959
Gutenberg	0.950	0.960	0.0103	0.00423	0.0277	2.61 × 10^−7^	0.909	0.944

The *Gini-like* *G*, the *Youth* *Y* and the *Recentness* *R* coefficients and the local entropy 〈*h*〉 computed for Last.fm, Github, Twitter, Wikipedia and Gutenberg. For comparison we report for each observable its values for the globally reshuffled sequences.

The *Youth* coefficient is defined by dividing *S* in intervals of length Δ*τ* and computing the average introduction time of the elements appearing in each interval, as a function of the interval index. If this curve grows fast it means that the trending elements are always new. On the other hand a slow growth indicates that old elements dominate. If we denote *λ* the slope of this curve (Fig 2 shows these curves for the non-textual data, while we refer to Fig C of S1 File for its representation for each of considered datasets), the coefficient *Y* is defined as $Y=\frac{\lambda }{\Delta \tau }$, bounded in [0, 1]. The Youth Coefficient quantifies how fast the system is renewed with successful new elements at each time step, so that *Y* = 1 indicates that all the elements in each interval are newly entered in the sequence, while *Y* = 0 indicates that all intervals mainly contain old elements.

The *Recentness* (of the Trending Elements) complements the information given by the *Youth* coefficient. Considering the same intervals of length Δ*τ*, we can consider the appearance time of the most popular element in each one, as in Fig 1. We compute the *R* metrics as the ratio between the sum of these appearance times over all the intervals and the value of this sum if the most popular element in each interval was the most recent possible. The Recentness quantifies whether the attention of the agents of the system is locally focused mainly on the first appeared elements (*R* close to 0), on the new ones (*R* close to 1), or shifts from one case to another (*R* ≈ 0.5).

The *Gini-like* coefficient, along as the *Youth* and the *Recentness*, characterise the distribution of popularity and its variation in time. We shall refer to them as “Variability of Popularity” metrics. While the *Gini-like* coefficient quantifies the effects of the waves of novelties from a global point of view (i.e., how they shape the global distribution of popularity), *Y* and *R* characterise their impact from a local perspective (i.e., how they contribute to the renewal of the sequence *S* in time and how the distribution of popularity is shaped when considering subsets of it). Finally, to quantify how clustered is the popularity of elements within each interval, i.e., whether it is shared by a very small group of elements or a balance exists among all the elements appearing in the interval, we define the *local entropy* 〈*h*〉 as the average normalised entropy of the popularity distribution in each interval. A value of 〈*h*〉 close to 0 indicates that the popularity is usually clustered around the most popular element, while a value close to 1 indicates a more uniform distribution. In all the datasets considered the value of 〈*h*〉 (see Fig C of S1 File) is almost constant in every interval, so that the average value is representative of the whole sample. Table 1 reports the values of *G*, *Y*, *R* and 〈*h*〉 for the datasets considered. For each metrics, we compare the values it takes on the data sequences with those of the corresponding globally reshuffled sequence *S*_*G*_. Overall, two different behaviours can be highlighted, one observed in the Last.fm, Twitter and Github datasets, where waves of popularity are more pronounced, and the other observed in textual datasets (Wikipedia and Gutenberg), where changes in popularity are less pronounced. The discussion for the textual datasets is presented in Section D of S1 File, where it is shown how the waves of novelties phenomena can be better highlighted in these data provided texts are preprocessed to remove stop words and reduced to their lexical components. More in details, though in all the datasets *G* > 0 indicates that there is some advantage in being an element introduced in the early stages of the dynamics, *G* is close to 1 only in the textual datasets, indicating that in Last.fm, Twitter and Github “young” elements also share a considerable part of the overall popularity. Similar results can be found also looking at *Y* and *R*. We found that the values taken by these observables in non-textual data clearly point to a rejuvenation of the system if compared with the values of the corresponding reshuffled sequences. For each system *Y* clearly indicates that there is a general growth of the first appearance time of popular elements in sub-intervals of the sequences. Thus the elements tend to become younger on average as the time grows, but note that the growth of the average appearance time of the elements visited by the system is rather slow: a value of *Y* < 0.5 indicates that the average age of the elements in each time frame is slightly biased towards old elements. *R* is close to 0.5, indicating that the most popular element in a sub-interval might be equally likely an old or a recent one. Note that for reshuffled sequences *R* ≈ 0, so that local success of young elements differs from global success and is due to the local clustering of elements of the same age, which is destroyed when such elements are randomly reordered. Finally, the general high value of the local entropy indicates that there is no strong dominance of a very popular element on all the others: for non-textual data 〈*h*〉 ≃ 1 indicating a quite homogeneous local distribution of popularity among the elements in each sub-interval. The emerging picture is that of systems where the dominance of the first introduced elements that achieve popularity is undermined by the appearance of novelties capable of gathering the attention of the public in local time frames. Locally the popularity is quite homogeneously distributed among recent and not recent elements and this fact can lead to the existence of “trending elements” that might be old as well as quite novel. While from a global perspective old elements are still dominant, i.e., being a firstly introduced elements is still an advantage, such advantage is diminished by considering a local temporal scale, where the global attention can shift over more recent elements.

### Modelling the expansion of and into the adjacent possible through exploration and exploitation

In [39] the dynamics of the adjacent possible has been modelled as a Polya’s Urn where the set of possibilities are represented by coloured balls in a urn and one constructs a sequence of coloured balls, i.e., the *actual history*, by sequentially drawing balls at random from the urn. The adjacent possible, i.e., the set of coloured balls never extracted before, and its expansion is introduced in this scheme by imposing that whenever one extracts a ball never observed before in the sequence, a certain number of brand new balls are added to the urn. From now onwards we shall refer to this model as Urn Model with Triggering (UMT). This modelling scheme succeeded in explaining the observations of the Zipf’s and Heap’s laws [43, 47] in several empirical datasets mirroring human activities. In addition, the introduction of a parameter representing the semantic correlation between elements in the urn, allowed to explain the existence of correlated innovation and their clustering in time [39]. However, this simple formulation of the model does not explain the existence of waves of novelties, since in this scheme the first elements introduced will always capture most of the overall attention. We now proceed to a generalisation of the original model to understand how new elements can emerge and diffuse in a population, sometimes becoming even more successful of the already established ones, despite the “rich-get-richer” mechanism characterising the dynamics of many real systems. To this end we introduce two new key ingredients meant to be able to control the expansion of the adjacent possible: (i) an adjustable bias between the choices of retracing the past or looking at the future; (ii) a collective effect in how the space of possibilities is carved. We shall refer from now onward this generalisation as GUMT. Despite their simplicity, these two ingredients turn out to be able to explain the emergence and evolution of waves of novelties.

**Fig 3:**
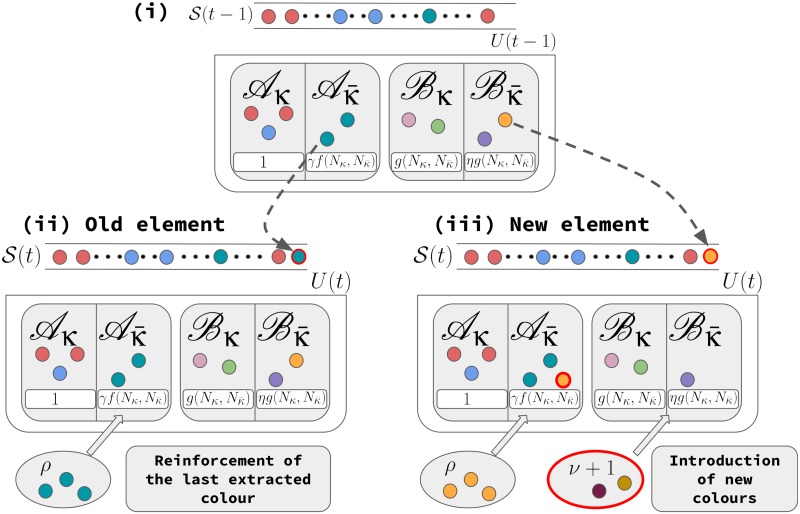
Pictorial representation of the model. Supposing that at time *t*−1 the last extracted ball has label *κ*, we can divide the urn into two large classes of balls: the class A including colours already appeared in *S* and the class B including colours that never appeared, and thus belonging to the adjacent possible, at time *t*. These two classes are further subdivided in two classes of balls sharing (*κ*) and not sharing ($\bar{\kappa }$) the label *κ*. The probability of extracting a ball at time *t* is proportional to a weight depending on the sub-class it belongs to. In particular the weights are 1 and $\gamma f(N_{\kappa },N_{\bar{\kappa }})$ for sub-classes $A_{\kappa }$ and $A_{\bar{\kappa }}$, respectively and $g(N_{\kappa },N_{\bar{\kappa }})$ and $\eta g(N_{\kappa },N_{\bar{\kappa }})$ for sub-classes $B_{\kappa }$ and $B_{\bar{\kappa }}$, respectively. (See the Methods section for details). If at time *t* an already appeared colour is extracted, then *ρ* balls with the same colour are added to the urn; if instead a ball is extracted from the adjacent possible, *ρ* balls with the same colour are added to the urn (now within the class of the ball already appeared) and *ν* + 1 balls of different colours sharing the same brand new label are added to the adjacent possible class.

The first ingredient is modelled considering at each time a higher probability to interact with each of the elements already visited by the system, i.e., already belonging the *actual history*, with respect to those living in the urn as future possibilities (refer to the Fig 3 and to the Methods section for a complete definition of the model). The second ingredient deserves a little discussion. In the UMT model it has been assumed that the collectivity of agents within a system can be described by a single entity, a sort of *average agent*, so that every agent explores and shares the same knowledge of the adjacent possible. Removing this constraint, i.e., considering instead a population of agents each exploring independently her own space of possibilities, can have very important consequences. First of all there is no more such a thing as a shared space of known experiences and a shared adjacent possible space. Each agent will have her own personal history and the overlap with the explorations made by other agents can be very small. This implies a breaking down of the whole space accessible to the population in regions presumably visited only by a limited number of agents. In this way each agent has only a limited visibility of the whole process at the population level. This heterogeneity of experiences and histories can be very important for the emergence of waves of novelties: the limited vision of the whole space might prevent the dynamics to head back to old and popular elements, allowing instead new elements to become more popular. With this rationale in mind, in our generalisation of the urn model we keep the simple structure of the *average agent* but we introduce a multi-agent effect in the form of a modulation of the accessibility of the space from each element based on the number of times it has appeared in the actual history (we refer for that to the Methods section, and in particular to the definition of functions *f* and *g*). From this mean-field perspective, highly visited elements are also supposed to be visited by a large number of agents and are thus the gates to access to a large part of the space. In Fig N of the S1 File it is shown how clear correlation exists between the number of different users that have interacted with an element and the total number of appearances of the element itself, hence providing support for the introduction of this ingredient.

**Fig 4:**
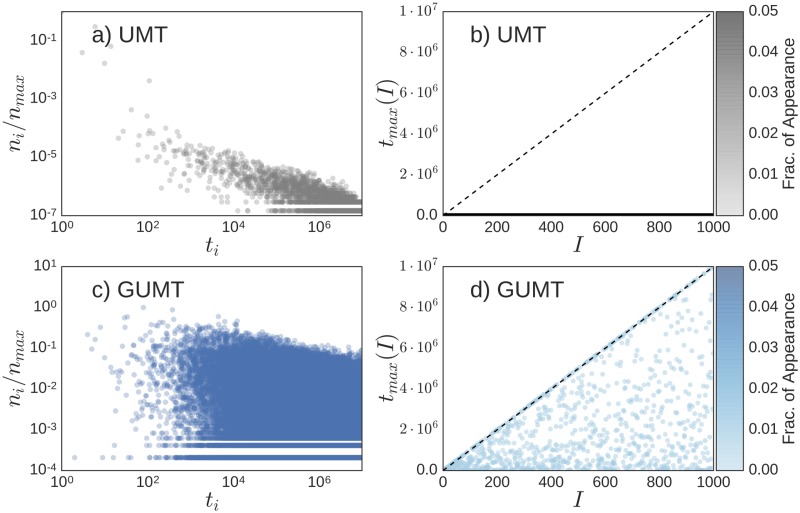
Comparison of the UMT and GUMT models. (Left-Panels) Frequency of occurrence *n*_*i*_ (suitably normalised with the maximum value *n*_*max*_) of each element *i* ∈ *S* as a function of its first appearance time *t*_*i*_. (Right-Panels) For each interval of length Δ*τ* we plot of the first appearance time of the most popular element within the interval, *t*_*max*_(*I*). The length of each interval is Δ*τ* = 10000. Top Panels are results coming from a simulation of the UMT model with parameters *ρ* = 2, *ν* = 2 and *η* = 0.4 for the model. Bottom Panels correspond to simulations of the GUMT model with *ρ* = 2, *ν* = 15, *η* = 0.001, *γ* = 0.004 and the choice II of the function *f* and *g*.

**Fig 5:**
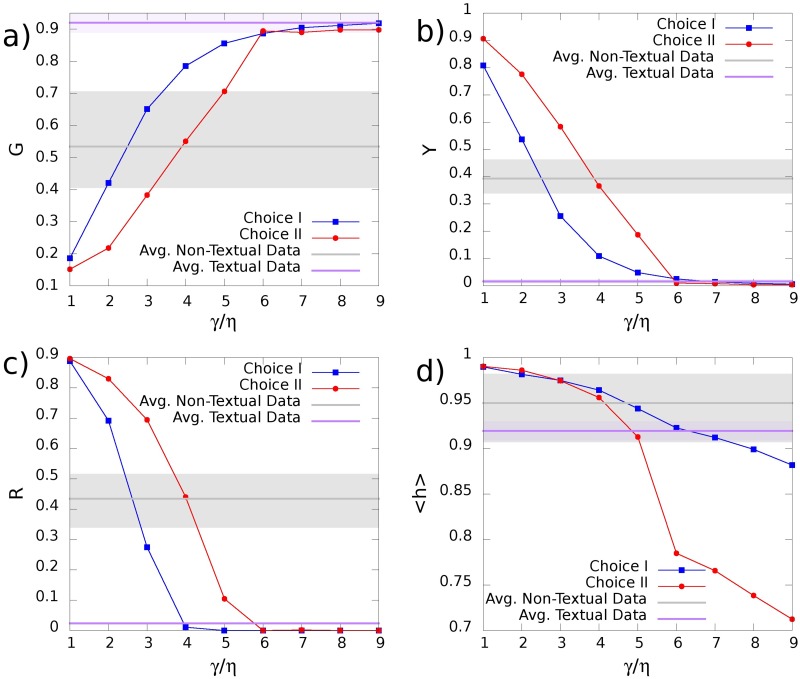
Values of the popularity metrics in the model for different values of the parameter *γ*. Numerical results for: (a) Gini-like coefficient *G*, (b) the Youth coefficient *Y*, (c) the Recentness *R* and (d) the local entropy 〈*h*〉, for different values of the ratio *γ*/*η* and the two choices of the function *f*. Blue lines correspond to results for the choice I of the function *f* regulating the time dependent connectivity of the graph with *ρ* = 1, *ν* = 500, *η* = 0.001, while red lines correspond to results the choice II of the function *f* with *ρ* = 2, *ν* = 15, *η* = 0.001 (see the Methods section for details). The horizontal black line in each panel indicates the average value of the presented popularity metrics measured on Last.fm, Twitter and GitHub datasets, while the highlighted gray area is the range of such metrics limited by the maximum and minimum measured values. The horizontal purple line represents the same average for the Wikipedia and Gutenberg dataset, being the highlighted purple area the range defined by the waves of novelties metrics on these two datasets.

We now show that the Generalised Urn Model with Triggering (GUMT) is able to reproduce, in addition to all the statistical signatures of the dynamics of novelties already highlighted in [39] (see Fig G and Section F of S1 File), both the global redistribution of popularity among recent and old elements and the local oscillations of success between old and new elements as highlighted in Fig 1 and summarised by the metrics introduced in the previous sections. Fig 4 shows the results for the same observables of Fig 1, namely the normalized frequency of occurrence *n*_*i*_ of each element *i* ∈ *S* as a function of its first appearance time *t*_*i*_ (Left Panels) and, for each interval of length Δ*τ*, the first appearance time, *t*_*max*_(*I*) of the most popular element within the interval *I* (Right Panels), both for the UMT and the GUMT models. It is evident how, thanks to the introduction of the two new ingredients described above, the GUMT is able to explain the same patterns of the most popular elements in different time-frames as observed in empirical data. Through Fig 5 we quantify this observation by showing the predictions of the GUMT framework for the four popularity metrics introduced above, namely the Gini-like coefficient *G*, the Youth coefficient *Y*, the Recentness *R* and the local entropy 〈*h*〉. We compare these predictions for several values of the model parameters, encoding the key ingredients (i) and (ii) defined above, with the average values of those observables in the empirical data. In particular in the *x*-axis of all the plots in Fig 5 is reported the ratio *γ*/*η*, modulating the propensity to exploit the past (high ratio) versus the propensity to explore the new (low ratio). When *γ* ≈ *η* we are in the case of (almost) linear growth for the Heaps’ law (refer to the Fig H in the S1 File). In this case all the popularity metrics indicate that both locally and globally the distribution of popularity is extremely uniform and there is a large bias towards new elements. In other words new elements appear at every time step but none of them is able to reach high popularity due to a high turnover: each new most popular elements is immediately replaced by a new one. When *γ* ≫ *η* we have instead the opposite case of slower growth of *D*(*t*) and all the metrics indicating a strong dominance of the old elements with respect to the recent ones, so that successful novelties never emerge. A more realistic behaviour can be found in intermediate cases, with *γ* > *η*. In this regime, in fact, novel elements can emerge and keep their popularity for enough time to be considered “successful”. This region of parameters is the one that intersects the area highlighted in Fig 5, reporting the values found for the reported metrics in the considered datasets. The interpretation of this result is that real systems feature a balance between the drive to “explore” the adjacent possible and the propensity to “exploit” and reinforce what is already known, resulting in the alternation of old and new elements in leading the scene.

## Conclusion

The emergence of new trends is a widespread phenomenon, affecting many fields of human activities. Despite its intuitive character, no satisfactory frameworks were available so far to quantitatively explain the occurrence of this phenomenon in its generality. Here we managed to fill this gap with a twofold approach: introduce suitable observables to empirically quantify the phenomenon and introduce a very general theoretical framework whose predictions are in good agreement with the empirical observations. In particular we introduced suitable metrics aiming at (a) identifying the unbalances in the distribution of the overall popularity between “old” and more recent elements introduced in a system, and at (b) identifying whether some of the elements are able to gain large popularity after their introduction, so that they end up being locally more popular than previously introduced elements. We tested these metrics in a series of datasets mirroring the dynamics of innovation and novelties in very different contexts of human activity. A very general empirical finding is that elements introduced at the early stages of the dynamics are in general more popular. Unlike what would be predicted by a simple rich-get-richer dynamics, it turns out however that old elements share their popularity with recently introduced elements, allowing in this way for the existence of waves of popularity. In local time frames, the most popular elements can be likely both old or recently introduced ones. This phenomenology is confirmed by looking both at local and global observables meant to quantify popularity.

In order to give a microscopic description of the above described phenomenology we introduced a theoretical framework inspired by the idea of adjacent possible, as initially introduced by Kauffman [37] and mathematically formalised in [39, 42, 48]. In particular the Urn Model with Triggering (UMT) introduced in [39] is able to reproduce the main stylised facts of the dynamics of novelties, such as the Heap’s and Zipf’s laws, as well as the presence of semantic correlations linking the occurrences of new elements. The original model introduced in [39] cannot however reproduce the empirical findings about the distribution of popularity, except in a narrow region of parameters where the Heaps’ law turns out to be linear and almost no semantic correlations exist among the elements of the system. To overcome this difficulty in this paper we presented a Generalised Urn Model with Triggering (GUMT) that introduces two new key ingredients, namely an adjustable bias between choices retracing the past or looking at the future and a collective effect on how the space of possibilities is carved. Semantic correlations are asymmetric in the sense that they are stronger for already visited elements than for elements not yet discovered. In this sense, we introduced a sort of inertia driving the dynamics towards the already known. The second ingredient introduces a modulation of the accessibility of the elements in the urn based on the number of times each element has appeared in the actual history. The rationale for this is the introduction of a multi-agent effect in a single-agent model. Larger is the number of occurrence of an element in the actual history, in fact, larger is presumably the number of individuals of a real population that explored that element and stronger will be the bias for future explorations of that element. The combined introduction of the two ingredients just discussed turns out to be able to accurately reproduce the patterns of popularity observed in the empirical data, as well as all the other observables like the asymptotic growth od *D*(*t*) and its relation with the frequency-rank distribution.

Finally, we point out that the presence of the asymmetry in the semantic correlations has a strong role in the emergence of the observed patterns of popularity. In particular, the inertia towards the already known must be not too strong, to prevent the dominance of old elements, nor too small, to prevent the system from moving always towards the new, hindering in this way the existence of successful elements. Real systems turn out to lie in between a pure *exploration*, i.e., the visit of brand new elements, and a pure *exploitation*, i.e., the retracing of the already known, strategies. This balance seems to be the key signature for the emergence of successful trends of recent elements, still preserving the existence of successful evergreens. The generality of our approach makes it suitable for the application in the most diverse contexts where people constantly struggle between the attraction for the new and the need to exploit existing findings. For these reasons we think this kind of approach could have a strong impact in all situations—education, research, technology, industrial competition, business—where innovation is unavoidable and exploitation is essential and a tradeoff is key for progress.

## Materials and methods

Here we define the dynamics of the Generalised Urn Model with Triggering (GUMT). Let us consider an urn U initially filled with *N*_0_ coloured balls, all of different colours, divided in $\frac{N_{0}}{\nu +1}$ groups such that the elements belonging to the same group share a common label *κ*. At time *t* = 0 all the balls in the urn share the same weight and the first ball is extracted from the urn randomly, with uniform probability. At each generic subsequent time step *t*, each ball in the urn belongs to one of the two classes: class A if the ball has been already extracted and thus appears in *S*(*t*−1) or class B, otherwise. Each of this class is further subdivided in two sub-classes depending on whether the balls share (*κ*) or not share ($\bar{\kappa }$) the label *κ*.

1. at each time step *t* we draw a ball from the urn with a probability proportional to its weight, depending both on the class of belonging (at time *t*), and on the label, say *κ*, of the ball extracted at time *t*−1, according to the following scheme:
  Class $A_{\kappa }$(balls with a colour already present in *S*(*t*−1) and carrying the label *κ*) and balls with the colour that triggered the introduction of balls with label *κ* in the urn (see point (iii) below): weight 1;
  Class $A_{\bar{\kappa }}$(balls with a colour already present in *S*(*t*−1) and not carrying the label *κ*): weight $\gamma f(N_{\kappa },N_{\bar{\kappa }})$, where *γ* ∈ [0, 1] is a constant and $f(N_{\kappa },N_{\bar{\kappa }})$ is a function of the number *N*_*κ*_ of balls in the urn that share the label *κ*, and of the number $N_{\bar{\kappa }}=N_{U}-N_{\kappa }$ of balls in the urn that do not share the label *κ*. $N_{U}$ is the total number of balls in the urn and we took implicit the time dependence of all these quantities. We choose *f* to be an increasing monotone function of *N*_*κ*_, bounded in [0, 1] (see below in the Methods section for its detailed definition).
  Class $B_{\kappa }$(balls belonging to the adjacent possible and carrying the label *κ*): weight $g(N_{\kappa },N_{\bar{\kappa }})$, where *g* is again an increasing monotone function of *N*_*κ*_ bounded in [0, 1].
  Class $B_{\bar{\kappa }}$(balls belonging to the adjacent possible and not carrying the label *κ*): weight $\eta g(N_{\kappa },N_{\bar{\kappa }})$, where *η* ∈ [0, *γ*] is a constant.
  If the ball *i* has been extracted at time *t*, then:
2. *i* is recorded in the sequence *S*(*t*), and put back in U along with *ρ* copies of it, where *ρ* is a positive real number;
3. If the colour of *i* was not already present in *S*(*t*−1) (i.e., it was drawn for the first time), we add *ν* + 1 balls with distinct colours not already present in the urn, all sharing a brand new label; we record that this new group was triggered by *i*, thus creating a “semantic connection” so that the new elements will have weight 1 if the last extracted ball as the same colour as *i*; similarly if one of the new elements is extracted, the weights of the same colour of *i* will have weight 1.

It is important to notice that the GUMT reduced to the UMT if we put *f* = *g* = 1 and *γ* = *η*. The above defined rules incorporate the two key ingredients of this modelling scheme: (i) an adjustable bias between choices retracing the past or looking at the future; (ii) a collective effect in how the space of possibilities is carved.

The asymmetry between the weights of the classes A and B implies a bias towards either the past or the future. On the other hand the specific form of *f* and *g* encodes the collective effect. More in particular, the functions *f* and *g* (increasing functions of *N*_*κ*_ bounded in [0, 1]) are applied to the weights of the balls; their purpose is to modulate the weights of these balls according to the total number of balls with label *κ*, reducing such weights whenever this number is small. In this way we model the fact that parts of the system that have not been visited frequently, are also those visited by a small number of agents of the population. Hence, the knowledge of the space is diminished leading to a smaller probability of visiting elements unrelated with the current one. In Fig N of S1 File we show that a clear correlation exists between the number of different users that have interacted with an element and the total number of appearances of the element itself, hence providing support for the introduction of this ingredient. Finally, the two parameters *η* < *γ* accounts for the fact that the probability of choosing an already extracted colour (an already known element) is higher than the probability of experimenting something completely new.

Both the above mentioned ingredients are necessary in order to reproduce the empirical patterns. In Fig I of S1 File S6 we show how the condition *γ* = *η*, (which is the case of the UMT model with the only addition of the functions *f* and *g*) leads to a behaviour similar to the UMT model. On the other hand, in Fig J of S1 File S6 we show how the model with the condition *f* = *g* = 1 and *η* < *γ* is able to predict the variability of popularity compatible with the empirical data only for extremely small values of *η* and *γ*, i.e., in a region of parameters where the Heap’s and Zipf’s laws are quite dissimilar from the empirically found ones.

### Choice of the functions *f* and *g*

We here define the functions $g(N_{\kappa },N_{\bar{\kappa }})$ and $f(N_{\kappa },N_{\bar{\kappa }})$, where *κ* indicates the label of the last extracted element, *N*_*κ*_ is the number of elements in the urn with this label and $N_{\bar{\kappa }}$ is the number of elements in the urn with a different label, in both cases including only the elements that have already appeared at least once in the sequence *S*. In section D of the S1 File, we show that when *f* = *g* = *const* (= 1), it is possible to derive upper and lower bounds for the exponent *β* of the growth of *D*(*t*); we also show that in the general case, in order to have the same asymptotic behaviour as in the case *f* = *g* = *const*, a general relation between the two functions must hold, namely $g\left(N_{\kappa },N_{\bar{\kappa }}\right)=\frac{N_{\kappa }+\gamma f\left(N_{\kappa },N_{\bar{\kappa }}\right)N_{\bar{\kappa }}}{N_{\kappa }+\gamma N_{\bar{\kappa }}}$ (see Section F of S1 File).

As for the specific choice of the function $f(N_{\kappa },N_{\bar{\kappa }})$ (and consequently $g(N_{\kappa },N_{\bar{\kappa }})$), we here adopt two possible forms:

- **Choice I**: $f\left(N_{\kappa },N_{\bar{\kappa }}\right)=\frac{N_{\kappa }}{N_{\kappa }+\gamma N_{\bar{\kappa }}}$, representing the ratio between the number of elements with the same label as the last extracted one, and the total number of elements in the urn (having been already extracted at least once), each with its own weight;
- **Choice II**: $f\left(N_{\kappa },N_{\bar{\kappa }}\right)=\frac{N_{\kappa }}{N_{\kappa }+N_{\bar{\kappa }}}$, representing the ratio between the number of elements with the same label as the last extracted one, and the total number of elements in the urn (having been already extracted at least once), unweighted.

Note that with both choices, every elements with a label so that $N_{\kappa }\gg N_{\bar{\kappa }}$ will have in $f\left(N_{\kappa },N_{\bar{\kappa }}\right)\approx g\left(N_{\kappa },N_{\bar{\kappa }}\right)\approx 1$ and hence the maximum possible access to the space.

## Supporting information

S1 File Supporting information file pdf file with detailed data description and analytical results. (PDF)
