# Supplementary material for: Waves of novelties in the expansion into the adjacent possible
Source: PLoS One. 2017 Jun 8;12(6):e0179303. doi: 10.1371/journal.pone.0179303 (PMC5464662; doi:10.1371/journal.pone.0179303)
Supplement: S1 File — (PDF) [file pone.0179303.s001.pdf]

Waves of novelties in the expansion into the adjacent possible.

Supporting Information File S1

Bernardo Monechi[1], Alvaro Ruiz-Serrano[1], Francesca Tria[2,1,*], Vittorio Loreto[2,1]

1 Institute for Scientific Interchange (ISI), Via Alassio 11C, 10126 Torino, Italy
2 Sapienza University of Rome, Physics Dept., Piazzale Aldo Moro 5, 00185 Roma, Italy
*** Corresponding Author: francesca.tria@roma1.infn.it**

A Datasets Information

Last.fm Dataset Last.fm is a music streaming service with a recommendation engine. Users can interact with the website listening to songs grouped by artist and album with the possibility of commenting and tagging them. The data used [1] contains the activity of 1000 until the 5th of May 2009. Each record of the dataset stores the song listened by a certain user, together with a timestamp and other information such as the user id and the artist that made the song. It contains 1.9×10^9 time ordered listened tracks, taken from a set of 1.5×10^6 different tracks.

Twitter Dataset Twitter is one of the most famous micro blogging social network which has been object of a vast number of studies within the scientific community. The dataset we used [2] has been collected every day in January 2013 recording the 10% of the whole users activity. Tweets without "hashtags" has not been considered along as the text of each tweet. The dataset is thus seen as a sequence of time ordered sets of hashtags adopted by users, each identified by her own id. Every hashtag is considered as a separate element, but we will not define an order for the hashtags within the same tweet. We restrict our analysis just to the first week of data, so in the end we consider 3.6×10^7 tweets containing 5×10^6 distinct hashtags.

Github Dataset Github [3] is a repository hosting service, which allows many software developers to work on a same project. Users can register to this online platform and create new projects or interacting with the already existing ones (provided they are public or they have access to them). The interaction can range from the creation of a new branch of the code, to the creation or modification of the Wiki page related to the project and so on. In our analysis we do not distinguish between the different possible interactions within the repositories and we focus instead on the time sequence of such interactions so that the elements of the time sequence are the repositories themselves. Moreover, even though it is possible to know whether an element appearing in the sequence for the first time was already present in the system or had just been created, we do make here this distinction considering the first appearance as the creation time. The data we used contains the activity of GitHub users in January 2015, with 1.4×10^7 actions performed over 1.5×10^6 repositories.

Wikipedia Dataset The English Wikipedia dataset [4] we analysed consists of 40000 different Wiki pages, randomly selected from the whole Wikipedia Corpus. The oldest timestamp of each page has been considered as the creation time of the page itself and we used this information in order to put the text of each page in a time ordered sequence of words. In the analysis we ignored capitalization and we considered words sharing the same root as different (e.g. *like* and *liked* are considered as different words). Moreover we considered homonyms as the same word, so that the past tense *saw* of the verb "to see" is the same word as the noun *saw*. The result is a unique text of 1.7×10^7 words, where the number of distinct words is 4.4×10^5. In order to decrease the contribution of the grammatical structure of the

texts and to focus on the lexical level, we excluded from each text 200 function words [5]. More details are shown in section D, where the effect the removal is discussed.

Gutenberg Dataset The Gutenberg Dataset [6] is the result of the crawling and filtering of data coming from the Gutenberg Project, performed during February 2007 and presented in [7]. The result is a collection of 4600 English texts of both prose and poetry. These texts contain a total number of 2.8×10^8 words, with 5.5×10^5 distinct words. In the same way as for the Wikipedia Dataset, we ignore capitalization, we consider words sharing the same root as different and we consider homonyms as the same word. In this case we do not have information about the time of creation of each text, thus we put them in a random order. As for the Wikipedia Dataset, we performed the analysis by removing function words.
All the dataset used for the analysis in this work can be downloaded from [8]. This data contains all the time series of each used dataset, where the elements (songs, words, hashtags, repositories) have been re-labelled with integers in order to ease the analysis.

B Statistical Signature of the dynamics of novelties and semantic correlations metrics

The dynamics of the appearance of novelties has been found to exhibit some statistical stylised facts across very different systems. In [7] two basic signatures have been identified and studied in various datasets mirroring human activities: the Heap's law [9], i.e., a sub-linear growth of distinct elements with the total number of elements, $D(t) = t^{\beta}$ with $\beta < 1$, and the Zipf's law, a power-law relation between the frequency of occurrence of a certain element and its rank (ranks are in descending order with the frequencies), $f(R) \sim R^{-\alpha}$ where f is the frequency and R is the rank. If one assumes a random-sampling from a set of elements with a power-law functional form of the frequency-rank distribution, a functional relation holds between the exponents β and α whenever β is smaller than 1, i.e. $\alpha = \frac{1}{\beta}$. Non trivially, this relation is also found empirically in the tail of the two distributions. A model of the emergence of innovations and novelties within a certain system should always consider these laws and their relation as the baseline for a correct reproduction of the empirical patterns. Following [7] we checked for the presence of the Heap's Law in each one of our datasets (see upper panels of figure A). We found that after a small transient in which D grows linearly, the growth becomes power-law like with an exponent β usually between 0.5 and 0.8. Note that in the Wikipedia Texts and Gutenberg cases the exponent is consistent with the one usually found for English texts. In the lower panel of figure A we report the Frequency-Rank distributions for the five datasets considered. In all cases we observe heavy-tailed distributions with a power-law tail whose exponent is consistent with the Heaps' law exponent highlighted in the upper panels.

In [7] it was also highlighted the presence of *semantic correlations* in the appearance of elements in time-ordered sequences corresponding to very different systems displaying innovation. Considering, in fact, each element of a system as belonging to a given semantic class (for instance a song belongs to a class defined by its author), new elements of the same class appear clustered in time, signaling the tendency of novelties to trigger the occurrence of other novelties. As an example, we may think to someone exploring the space of literature by reading different books. The phenomenon of semantic correlation would predict that, after having discovered and loved a specific author or a new genre, our reader will preferably look for book within the same semantic domain. The presence of semantic correlations has been quantified in [7] through two distinct methods, both resulting in the tendency of semantically related elements to appear clustered in time-ordered sequences of novelties. Considering each element in a time-ordered sequence $\mathcal{S}$ as belonging to a certain semantic class identified by a label, say "A", the first method consist in the computation of the distribution of the temporal intervals between the occurrences of two *new* elements with the same label, $f(l)$, both in the original sequence and in a globally reshuffled sequence, obtained by randomly permuting the elements of the original sequence. The second method consists in the computation of the average entropy, $S(k)$, of all occurrences of new elements whose common label "A" appears k times in $\mathcal{S}$ and its comparison with the same observable measured on a reshuffled $\mathcal{S}$. For each label "A" appeared k times in $\mathcal{S}$, one divides the sequence in k equal intervals and for each interval i one counts the number of times "A" appears in it. Indicating this number as

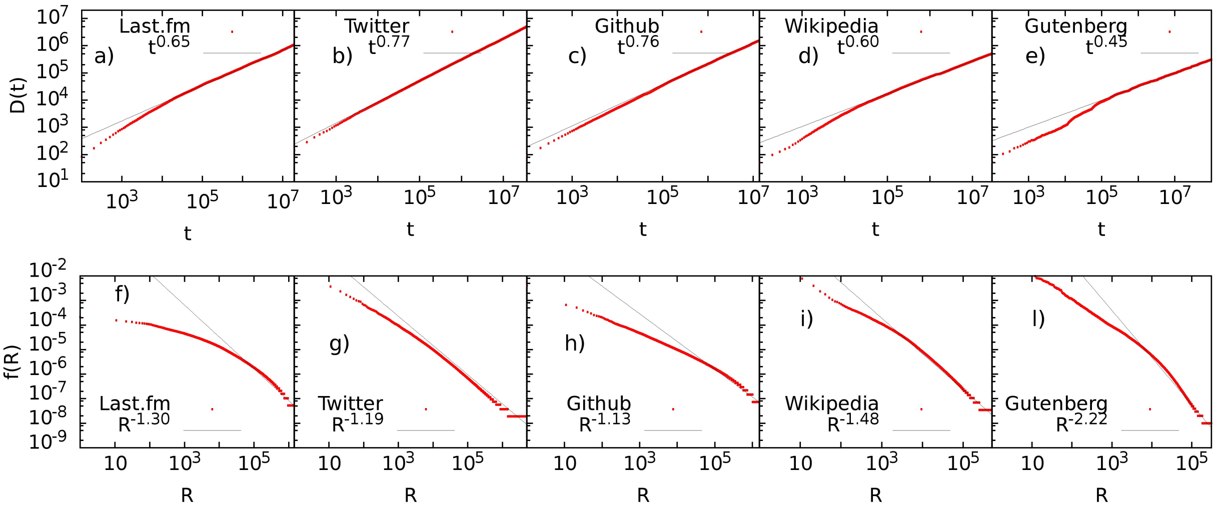

Figure A: **Heap's and Zipf's Laws** Heap's Laws for the Last.fm (a), Twitter (b), Github (c), Wikipedia (d), Gutenberg (e). Zipf's Laws for the Last.fm (f), Twitter (g), Github (h), Wikipedia (i), Gutenberg (l). Continuous lines correspond to power-law fits of the data.

f_i, one defines $S_A(k) = -\sum_i f_i/k \log(f_i/k)$. By averaging over all the labels appeared k times one obtains the average entropy $S(k)$. Note that $S(k) \in [0, \log k]$, so that $S(k) = 0$ indicates a very high degree of clustering while $S(k) = \log k$ indicates that clustering is not present. In order to compare different values of k, we rescale $S(k)$ by $\log k$ so that $S(k) \in [0, 1]$ for every k. The identification of a label for each distinct element in $\mathcal{S}$ is not an easy task in many databases. While for the Last.fm one, we can assume that the label of a song corresponds to the artist or the band that composed it, in the other cases this association if more difficult and the zeroth-order approximation is that each element has its own label. Note that this choice could lead to an underestimation of the degree of semantic correlation of the system, but nonetheless our aim here is just that of assessing their presence. Figure B shows the dependence of $\frac{S(k)}{\log k}$ on k (left panels) and the distributions $f(l)$ (right) for the Twitter, Wikipedia, and Github datasets. In all cases the comparison with the same observables computed on globally reshuffled sequences is presented. Having defined $S_G(k)$ as the entropy $S(k)$ computed on reshuffled sequences, we may introduce a way to quantify the difference between $S(k)$ and $S_G(k)$ as follows. Since both $\frac{S(k)}{\log k}$ and $\frac{S_G(k)}{\log k}$ are convex functions of k bounded in $[0, 1]$, their difference can be synthesised as:

$$\delta S = \frac{\sum_{k=1}^{k_{max}} (S_G(k) - S(k))}{\max\{\sum_{k=1}^{k_{max}} S_G(k), \sum_{k=1}^{k_{max}} S(k)\}}, \tag{1}$$

where k_{max} is the maximum value of k found in the dataset. This measure represents the ratio between the area between $S_G(k)$ and $S(k)$ and the maximum between the area under the curve of $S_G(k)$ and $S(k)$. Note the $\delta S \in [-1, 1]$, so that a positive value of δS indicates that the clustering of the labels is higher with respect to the random case and vice versa for $\delta S < 0$. In particular we found the following values for the datasets considered:

- $\delta S = 0.173$ for the Last.fm Database,
- $\delta S = 0.143$ for the Twitter Database,
- $\delta S = 0.063$ for the Github Database,
- $\delta S = 0.089$ for the Gutenberg Database,
- $\delta S = 0.085$ for the Wikipedia Database.

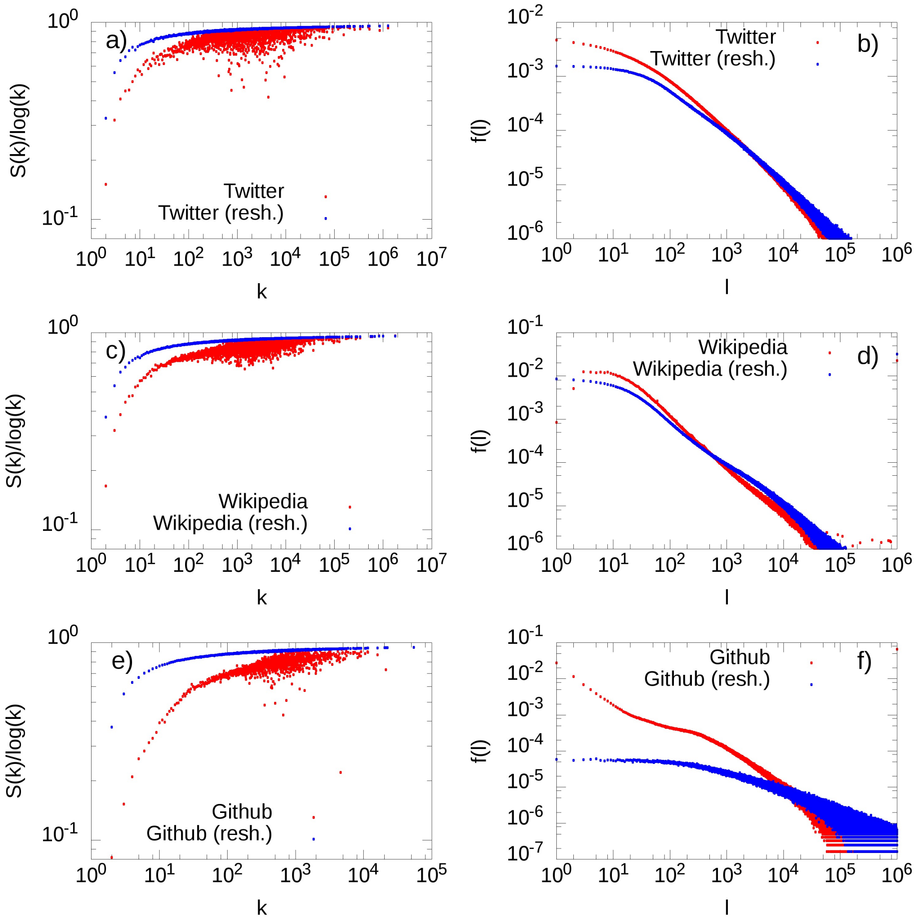

Figure B: Entropies for the Twitter, Wikipedia and Github (a-c-e) datasets. Distribution of the temporal distance between elements with the same label for the Twitter, Wikipedia and Github (b-d-e) datasets. Blue dots correspond to the same observables computed on globally reshuffled sequences.

C Waves of Novelties Metrics and Dependence with Δt

The sub-linear growth of the number of different novelties, the related power-law dependence of the Frequency-Rank distribution and the presence of semantic correlations are not the only signatures we detected in the dynamics of innovations. We have observed in the main text that a competition exists between "old" elements that dominate and polarise the attention of a given community and "recent" elements struggling to achieve popularity. This peculiar dynamics results in a weakening of the dominance of the older elements when the global sequence $\mathcal{S}$ is considered. When restricting to smaller time-frames, we observe that newer elements might be dominating over older ones so that the most popular element in a given time frame could be a recently introduced element. In order to quantify this phenomenology we have introduced several observables aiming at characterizing the distribution of the popularity of the elements of $\mathcal{S}$ in relation to their age in the system, looking at $\mathcal{S}$ both from a "global" and a "local" (in time) point of view. Here are the observables:

- **Gini-like Coefficient** G**:** we rank each element i of $\mathcal{S}$ according to its appearance time and we assign to each one of them two coordinates (x_i, y_i) in a Cartesian plane so that $x_i = \frac{r_i}{D}$, where r_i is the rank of i and D is the total number of distinct elements in $\mathcal{S}$, and $y_i = \sum_{j:r_j \leq r_i} f_j$, where f_j is the frequency of occurrence of the element j in $\mathcal{S}$. The points (x_i, y_i) define a curve in $[0,1] \times [0,1]$. The Gini-like coefficient G is defined as

the area in between the $x = y$ line and the (x_i, y_i) curve (Fig. C reports the planes (x_i, y_i) corresponding to the datasets we considered), i.e., the difference between the area, $A_{(x=y)}$ below the $x = y$ line and the area, $A_{(x_i,y_i)}$ below the (x_i, y_i) curve:

$$G = \frac{A_{(x=y)} - A_{(x_i,y_i)}}{A_{(x=y)}}. \tag{2}$$

The aim of the Gini-like Coefficient is to quantify how the popularity is shared among elements of different ages without focusing on a particular period of time. A coefficient of 0 indicates a uniform distribution of popularity while a value close to 1 signals a strong dominance of old elements.

- **Youth Coefficient** Y**:** Dividing $\mathcal{S}$ in $T/\Delta\tau$ sub-intervals of length $\Delta\tau$, for each interval I_i (with $i = 1, ..., T/\Delta\tau$) we compute the quantity:

$$\langle t \rangle_{I_i}(\Delta\tau) = \frac{1}{\Delta\tau} \sum_{j \in I_i} n_j t_j \tag{3}$$

  where n_j is the number of times the element j appears in I_i and t_j is the time of its first appearance in $\mathcal{S}$. As shown in Fig. C, in empirical data the relation between $\langle t \rangle_{I_i}$ and and the ordered sequence of I_i is roughly linear. Indicating with λ the slope of this relation, the Youth Coefficient is defined as $Y = \lambda/\Delta\tau$.

- **Recentness (of the Trending Elements)** R**:** Indicating with $\tau_i(\Delta\tau)$ the first appearance time of the most frequent element in I_i, we define the Recentness $R(\Delta\tau)$ as the ratio between the sum of $\tau_i(\Delta\tau)$ over all the intervals I_i and the sum of the maximum introduction time $(i+1)\Delta\tau$ that an element in each I_i can have:

$$R(\Delta\tau) = \frac{\sum_{i=0}^{\frac{T}{\Delta\tau}} \tau_i(\Delta\tau)}{\sum_{i=0}^{\frac{T}{\Delta\tau}} (i+1)\Delta\tau} = \frac{2\Delta\tau \sum_{i=0}^{\frac{T}{\Delta\tau}} \tau_i(\Delta\tau)}{(T+\Delta\tau)(T+2\Delta\tau)}. \tag{4}$$

- **Local Entropy** $\langle h \rangle(\Delta\tau)$ Considering the frequency of occurrence of each element $j \in I_i$, $f_j = n_j/\Delta\tau$, we can compute the normalized entropy of the discrete probability distribution given by them:

$$h_i(\Delta\tau) = \frac{-\sum_{j \in I_i} f_j \log f_j}{\log D(I_i)}, \tag{5}$$

  where $D(I_i)$ is the number of distinct elements in the interval I_i. Fig. C shows that h_i is quite constant in every interval and hence the average of the entropy $\langle h \rangle(\Delta\tau)$ over all the intervals is representative of the typical local situation.

Table A reports the values of all the observables defined so far for all the datasets considered. As for the Gini-like coefficient we observe a positive value for all the datasets considered, indicating that the first introduced elements in $\mathcal{S}$ are dominating. However, this value for non-textual data is far from 1 signaling a situation where old and new elements constantly compete for popularity. The Youth Coefficient quantifies if and how fast the average first appearance time of the elements in a certain period of time grows in time. We checked that in every non-textual dataset this coefficient indicates a "rejuvenation" of the elements so that newer ones come into play in statistically significant numbers, able to lower the average age of the elements in each time interval. The Recentness instead quantifies how likely is that the most popular element in a time-frame is either old or new. The value of about 0.5 found in non-textual datasets indicates that there is not a defined pattern in the age of such element: in each time-frame it can be very recent, old or in between. Finally, the local entropy, which is basically the normalised entropy of the distribution of popularity in a time interval of length $\Delta\tau$, quantifies if this distribution is uniform or clustered around the most popular element. Since, for every dataset the local entropy is very close to 0.9 we can argue that the local distribution of popularity is quite uniform and the most popular elements are not strongly dominating above the others.

Three of these metrics where actually depending on $\Delta\tau$, i.e., the width of the bins in which we divided the global time sequence of elements. In Fig. D we show these dependencies measured with all the datasets introduced in the

	G	G (resh.)	Y	Y (resh.)	R	R (resh.)	$\langle h \rangle$	$\langle h \rangle$ (resh.)
Last.fm	0.491	0.685	0.379	0.056	0.516	7.46×10^{-6}	0.982	0.997
Twitter	0.405	0.628	0.463	0.089	0.448	5.24×10^{-5}	0.961	0.993
GitHub	0.706	0.85	0.339	0.128	0.386	2.34×10^{-6}	0.907	0.945
Gutenberg (with func.)	0.997	0.982	0.0047	0.0.0015	1.903×10^{-5}	1.69×10^{-7}	0.796	0.804
Gutenberg	0.950	0.960	0.0103	0.00423	0.0277	2.61×10^{-7}	0.909	0.944
Wikipedia (with func.)	0.926	0.938	0.0235	0.0145	2.33×10^{-6}	1.47×10^{-6}	0.830	0.841
Wikipedia	0.889	0.907	0.035	0.022	0.02	1.78×10^{-6}	0.930	0.959

Table A: **Variability of Popularity metrics** The *Gini-like* G, the *Youth* Y and the *Recentness* R coefficients and the average entropy $\langle h \rangle$ computed for Last.fm, Github, Twitter, Wikipedia and Gutenberg. For comparison we report for each observable its values for the globally reshuffled sequences. For Wikipedia and Gutenberg we report the results obtained with and without the removal of function words.

main text. We see that the Youth Coefficient is independent from $\Delta\tau$ for all the datasets, while the Youth Coefficient shows a weak dependence on $\Delta\tau$ only for the Last.fm dataset. Though the local entropy displays a logarithmic decrease with $\Delta\tau$, this does not affect the conclusions we draw based on this observable.

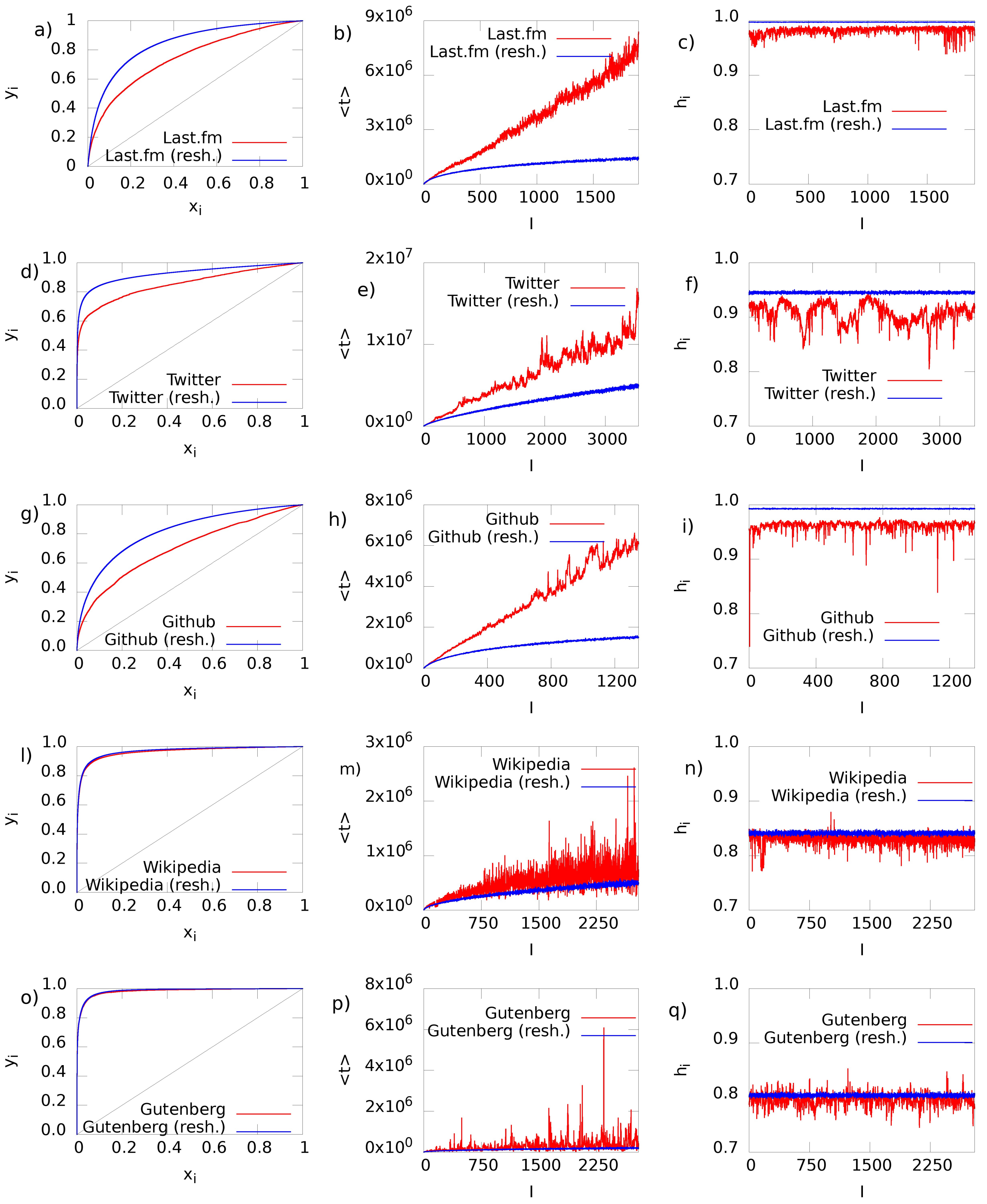

Figure C: **Popularity metrics and Waves of Novelties**. Planes (x_i, y_i) to define the Gini-like coefficient G (left column), graph to compute the Youth Coefficient Y (central column) and local entropy $\langle h \rangle(\Delta\tau)$ (right column) for the Last.fm (a-b-c), Twitter (d-e-f), Github (g-h-i), Wikipedia (l-m-n) and Gutenberg (o-p-q) datasets. In the x-axes of the central and right columns we denote (with a little abuse of notation) with I the (ordered) index denoting each sub-interval.

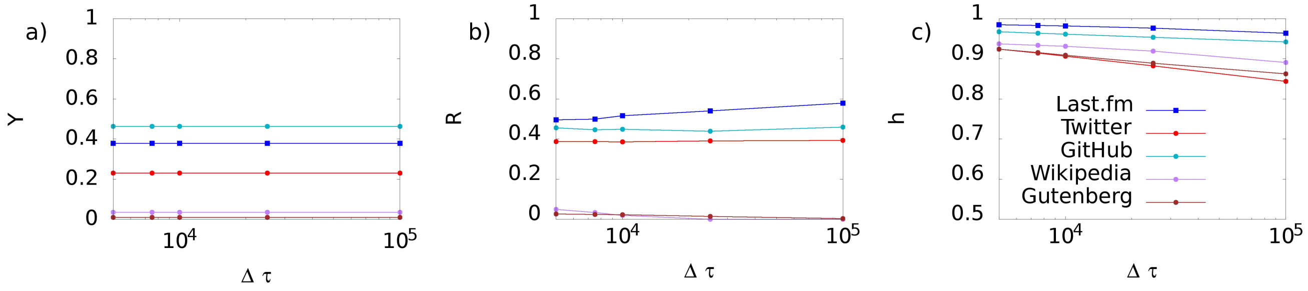

Figure D: (a) Youth Coefficient Y, (b) Recentness R and (c) Local Entropy $\langle h \rangle$ as a function of the bin length $\Delta\tau$ for all the databases introduced in the main text.

D Waves of Novelties for Textual Data

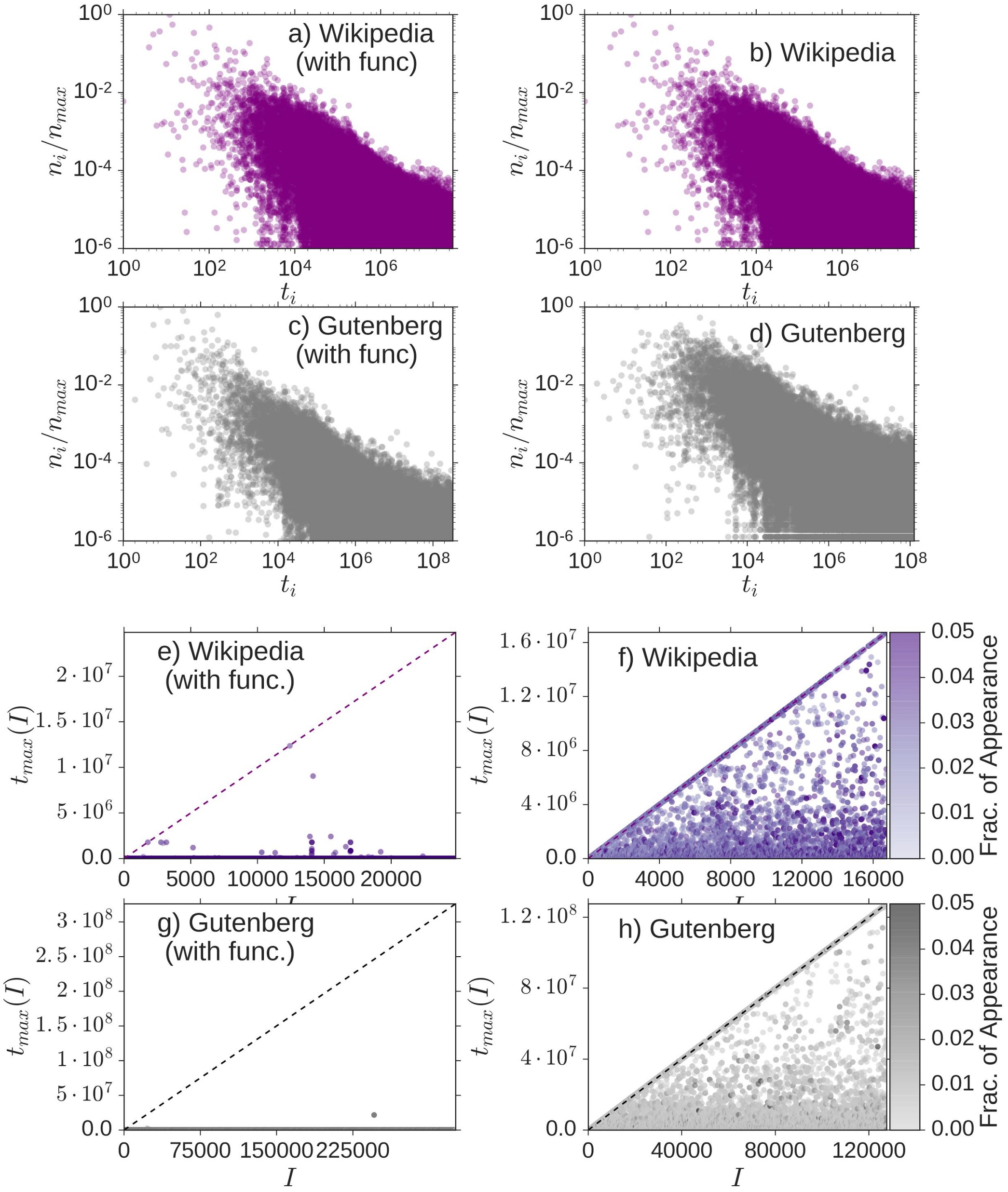

Figure E: **Waves of Novelties in Texts.** These plots are the same as in Fig. 1 of the main-text, for textual data. (a-b-c-d) Normalised frequency of occurrence n_i of each element in $i \in S$ as a function of its first appearance time t_i for the Wikipedia (a-b) and Gutenberg (c-d). (e-f-g-h) First appearance time of most popular element per each interval for the Wikpedia (e-f) and Gutenberg (g-h) datasets. Left panels show the results for the whole datasets, while right panels show the same results for the datasets where we excluded function words. Here the length of the interval is $\Delta t = 1000$.

The analysis related with the phenomenon of Waves of Novelties requires a correct temporal ordering of the elements of each considered datasets. For textual data, we are able to correctly identify the temporal sequence of the texts just in the Wikipedia dataset since the creation time of each Wikipedia page is clearly indicated in the data. Hence we can create a time sequence S of words by joining the texts of the pages following these creation times. Unfortunately the same situation does not apply to the Gutenberg case. Gutenberg corpus, in fact, do not systematically provide a time stamp for each book. For the sake of our analysis we created a randomly ordered sequence of the Gutenberg texts. This implies that we keep the ordered structure within texts and we neglect higher correlations across texts. As already mentioned in the main text, we pretend that the this procedure does not affect our conclusions as confirmed by the similarity of the results obtained for Wikipedia case as seen in Fig. E (panels a,b,c and d). We can notice, in fact, that the patterns linking n_i and t_i observed in Fig. 1 of the main-text are precisely the same both in the case of the random sequencing of the books of the Gutenberg corpus and of correctly time-ordered texts of Wikipedia. In Fig.1 of the main text we have shown that when the sequence $\mathcal{S}$ is divided into subintervals of length $\Delta\tau$, the most popular element in each time frame the popularity is shared among old and recently introduced elements. It is interesting to look at this phenomenology in textual data. In textual data, as one can easily guess, the emergence of waves of popularity is strongly affected by the underlying syntactic and grammatical structure. In fact, one can expect that in a text there will be variability in the frequency of occurrence of the topics treated, so that the first introduced topics are not always dominating through the whole text, but new ones are introduced from time to time and might dominate the discussion for a while. On top of this is the grammatical structure, which does not really change throughout the text: non-lexical words (i.e., words without semantic meaning) like conjunctions (”and”, ”or”), articles (”the”, ”a”) or auxiliary verbs will be the most frequent words used in each subinterval. In order to separate these two effects we performed an experiment by removing from textual data a set of 200 function words [5]. In order to compare the effects of this removal, in Fig. E panels e,f,g and h we show the same plots of the left panels of Fig. 1 of the main text, for the textual datasets with and without function words. We can see that in the first case, the first introduced elements are the ones that are always dominating while in the second one there is more variability. This is due to the fact that function words are clearly among the first words to be introduced and are always the most frequent words in each sentence since they are used to connect lexical words and build the grammatical structure. Removing such elements allows the lexical part to emerge, hence we see that also recently introduced words can be locally dominating. This is also evident if one compares the values of the popularity metrics reported in Table A for the textual datasets with and without function words. The Gini Coefficient is larger than 0.9 indicating a high dominance of the firstly introduced elements. $R(\Delta\tau) \approx 0$ and Y indicates that there is no growth on the average appearance time in sub-interval and the most popular element is always an old one. The local entropy indicates a higher degree of clustering of the popularity with respect to the other datasets, but its value is still close to 1 indicating that the scenario is probably an almost uniform distribution with few highly dominant elements. By removing the set of 200 function words, the scenario slightly changes. There is a clear shift in the Gini Coefficient towards smaller value indicating that the global predominance of recent elements has increased. Concerning the local metrics, Y and R are slightly higher. Moreover R is now not consistent with the random case and the local entropy takes values closer to the ones found for the other datasets. We speculate that reducing each text to its lexical components could align the computed metrics to the values of the other datasets. In this case, in fact, we recover a picture similar to those observed in non-textual datasets, providing to keep the interval $\Delta\tau$ an order of magnitude smaller than for non-textual datasets. Increasing it by a factor 10 will destroy the observed pattern again, leading to a dominance of old elements in each interval. Thus, it is likely that the texts should be cleaned also from other constructs, such as frequent idiomatic expressions. A further development could be the aggregation of words according to a particular semantic area, that could be performed used well-known tools in natural language processing such as the Latent Dirichlet Allocation (LDA) [10].

E Urn Model with Triggering (UMT)

The statistical patterns connected with the waves of novelties coexist, in all the datasets considered (see main text and section D), with the other statistical signatures of the emergence of novelties discussed in section B. Hence, the mathematical framework of the adjacent possible expansion introduced in [7] seems to be the ideal one to reproduce this phenomenology. In this section we briefly introduce that framework and we show that the model introduced in [7] is suitable to reproduce the values of the popularity metrics observed in empirical data only when the growth of the

number of distinct elements $D(t)$ is linear. We have seen from the values of δS in section B, that semantic correlations are always present in each dataset. Hence, we will use the version of the model in which semantic correlations are present, referring to it as "Urn Model with Triggering" (UMT). In this model, coloured balls are extracted from a urn $\mathcal{U}$ representing a growing space of possibilities (ideas, cultural or artistic artefacts, etc,). Whenever a ball is extracted, a certain number of balls of the same color are put in $\mathcal{U}$, increasing the probability that the same color will be extracted again. In order to model the expansion of the Adjacent Possible, whenever a colour is extracted for the first time, a certain number of brand new colours is injected in $\mathcal{U}$ making the space to expand. Finally, the semantic relation between elements in the urn is introduced through labels attached to each ball. In this scheme elements with the same label are semantically related. When a ball with a given label is extracted, the probability of extracting a ball with a different label is rescaled through a parameter $\eta < 1$ that quantifies the strength of semantic correlations (the smaller such parameter, the stronger the correlations are).
More in detail, we recall that the model starts with an initial number of elements N_0 in a urn $\mathcal{U}$, divided in $\frac{N_0}{\nu+1}$ groups with different labels assigned with $\nu \in \mathbb{N}$. At each time step a ball is extracted and put into a time-ordered sequence $\mathcal{S}$. At each time t:

(i) one extracts a ball i from $\mathcal{U}$ with a probability proportional to its weight w_i, which is assigned according to the ball extracted at time $t-1$ (say j):

  (a) $w_i = 1$ if the ball has the same label as j.

  (b) $w_i = \eta$ if the ball has not the same label as j, where $\eta \in [0, 1]$.

(ii) One adds $\rho \in \mathbb{N}$ balls of the same color of i in $\mathcal{U}$ and one records i in the sequence $\mathcal{S}(t)$.

(iii) If i appears for the first time in $\mathcal{S}(t)$, $\nu + 1$ brand new balls of distinct colours, all sharing a same brand new label, are added to $\mathcal{U}$.

In this model, a ball is extracted with a probability proportional to its weight, depending on the number of times the ball has been extracted before and since after each extraction ρ balls with the same colour are put in $\mathcal{U}$, a reinforcement mechanism is at play. The expansion of the adjacent possible is modelled through the introduction of the $\nu + 1$ balls with brand new colours as in step (iii). The presence of semantic correlations is modelled through the labels attached to the balls and through the parameter η, which reduces the probability of extracting a ball with a label different from the previously extracted one. It has been shown that in this model the exponent of the Heap's Law is bounded in $[\min(\frac{\nu\eta}{\rho}, 1), [\min(\frac{\nu}{\rho}, 1)]$ and it has been shown numerically that the exponent of the Zipf's Law corresponds to $\frac{1}{\beta}$.
Since in our datasets the exponent of the Heap's law is always larger than $t^{0.5}$, in the following study we fix the lower bound of β keeping the ratio $\beta_0 = \frac{\nu\eta}{\rho}$ constant. Starting from a large value of η we study the behaviour of the model as η decreases in order to check whether the waves of novelties might be triggered by the local constraints in the dynamics induced by the semantic correlations. The results are shown in figure F for two chosen values of β_0. With these choices, we see that as far as η is large enough, we have a sub-linear growth of $D(t)$ but all the other popularity metrics are off with respect to the empirically observed values. When η is sufficiently small the popularity metrics introduced above starts to indicate variability, though the growth of the number of distinct elements is linear, or almost linear, in time. The linear and sub-linear growth regions are divided by a vertical line in every panel of figure F. We can see that the local entropy is never larger than 0.9 in the region of sub-linear growth and R is strictly equal to 0 in complete disagreement with the empirical findings. Concerning G and Y it is possible to find values different from 0 and 1 respectively in the sub-linear growth region, but values compatible with the measures performed on our datasets emerge just in the case of a linear growth for $D(t)$. This implies that, in order to match all the empirical findings for the emergence of waves of novelties, some refinements in the modelling scheme are in order.

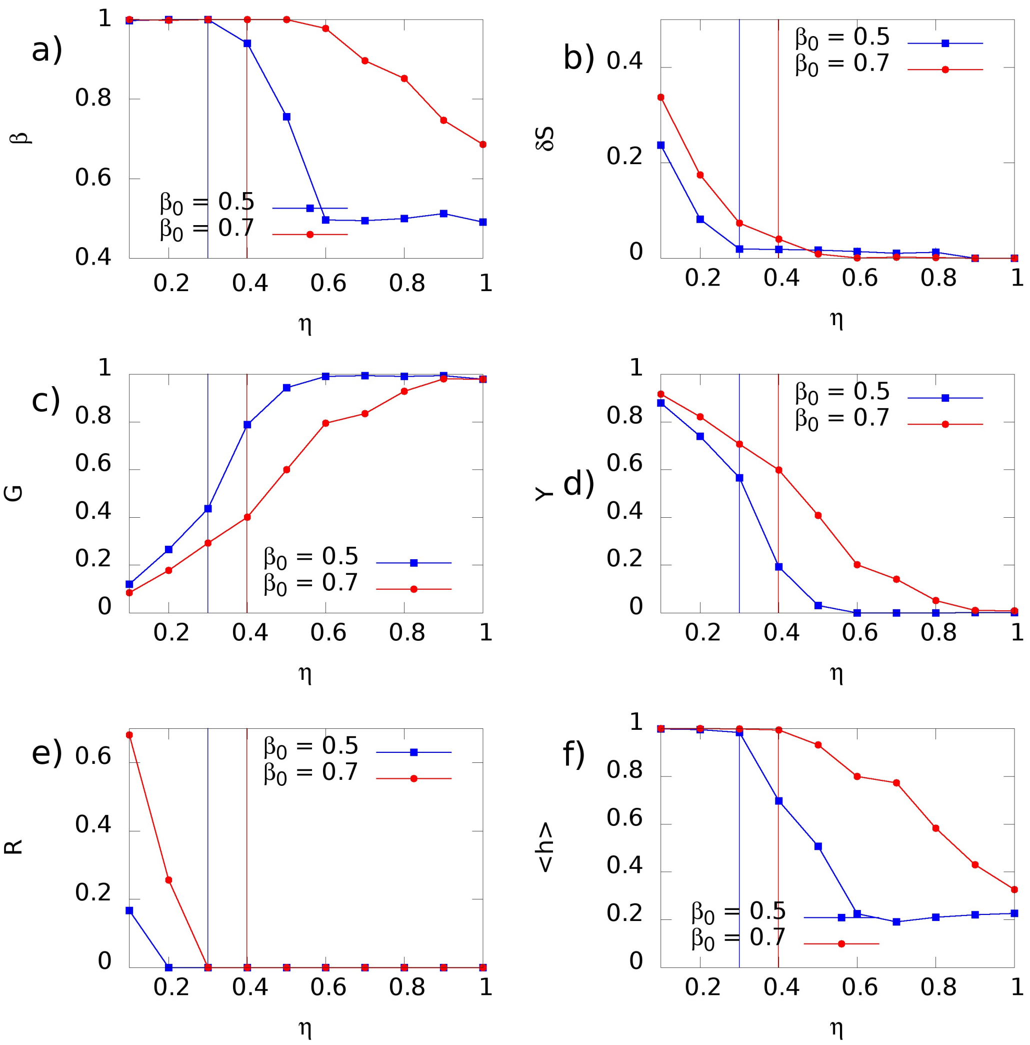

Figure F: **Adjacent Possible Expansion Model with Semantic Correlations** Numerical Results for the model introduced in [7] with $\beta_0 = 0.5$ (red line) and $\beta_0 = 0.7$ (blue line). Heap's law growth exponent β (a), δS (b), G (c), Y (d), R (e), $\langle h \rangle$ (f) as functions of the semantic correlations parameter η. Red and blue vertical lines indicates the largest value of η for which $\beta \neq 1$ for $\beta_0 = 0.5$ and $\beta_0 = 0.7$ respectively. All the simulations have been performed for 2.5×10^6 time steps.

F Generalized Urn Model with Triggering (GUMT)

In sections E, we showed that the UMT is not capable of reproducing the patterns related with the waves of novelties according to the popularity metrics introduced in section C. In order to generalise the model and make it suitable to reproduce such patterns, we introduced two new ingredients, representing the “inertia” in the exploration of the new and the collective effects of the community exploring the space of novelties. We refer to the related Results section of the main text for a detailed discussion of the meaning of these two ingredients. In section H, we give a small justification of the introduction of the collective effect of the community by showing the strong correlation between the number of users that have visited or interacted with an element and the frequency of occurrence of the elements itself.

Here, we formally introduce the model and present some analytical results related to the growth of the number of distinct elements at time t, say $D(t)$. Since two different ingredients are introduced, we discuss the effects of each of them separately, showing how their interplay is necessary in order to correclty reproduce the emergence of the waves of novelties.

As in the UMT model, here we have a urn $\mathcal{U}$ with initially N_0 balls, divided in $\frac{N_0}{\nu+1}$ groups each sharing the same label κ. At first a ball is drawn with uniform probability and ρ copies of it added to the urn. We keep track of the sequence of the extracted balls until time t in $\mathcal{S}(t-1)$. At each time step t we extract a ball i from $\mathcal{U}$ with a probability proportional to a weight and we add ρ copies of it to $\mathcal{U}$. If the choses never appeared in $\mathcal{S}(t-1)$, the appearance of this novelty triggers the introduction of $\nu+1$ balls with brand new colours in the urn sharing the same brand new label. Note that all the balls with colours that are not present in $\mathcal{S}(t-1)$ are considered in the Adjacent Possible and hence the appearance of a novelty causes the expansion of the Adjacent Possible itself. The weights for the extraction of a ball depends on the fact that the color of the ball is already in $\mathcal{S}(t)$ and on whether the label of the ball is the same as that of the last extracted ball. Supposing that the last extracted ball has label κ, we indicate with N_κ the total number of balls sharing the same label whose colour has already appeared in $\mathcal{S}(t-1)$. We indicate with $N_{\bar{\kappa}}$ the number of balls with a different label and a colour already appeared in $\mathcal{S}(t-1)$. We thus divide the balls in $\mathcal{U}$ in 4 different classes, based on their label and on whether they to the Adjacent Possible. The weight used to compute the probability of extracting a ball depends on its class. In particular (refer to Fig.2 of the main text):

- If the ball has already appeared in $\mathcal{S}(t-1)$ and shares the same label κ of the last extracted ball, then it belongs to the class $\mathcal{A}_\kappa$ and its weight is 1.
- If the ball has already appeared in $\mathcal{S}(t-1)$ and shares a label different from κ, then it belongs to $\mathcal{A}_{\bar{\kappa}}$ and its weight is $\gamma f(N_\kappa, N_{\bar{\kappa}})$, where $\gamma \in [0,1]$ and $f(N_\kappa, N_{\bar{\kappa}})$ is an increasing function of N_k (and hence decreasing in $N_{\bar{\kappa}}$) bounded in $[0,1]$.
- If the ball has never appeared in $\mathcal{S}(t-1)$ and its label is κ, then it belongs to the class $\mathcal{B}_\kappa$ and its weight is $g(N_\kappa, N_{\bar{\kappa}})$ where $g(N_\kappa, N_{\bar{\kappa}})$ is an increasing function of N_k (and hence decreasing in $N_{\bar{\kappa}}$) bounded in $[0,1]$.
- If the ball has never appeared in $\mathcal{S}(t-1)$ and its label is not κ, then $\mathcal{B}_{\bar{\kappa}}$ and its weight is $\eta g(N_\kappa, N_{\bar{\kappa}})$, where $g(N_\kappa, N_{\bar{\kappa}})$ is the same of the previous point and $\eta \in [0, \gamma]$.

The parameter $\gamma > \eta$ is there to capture the idea that it is easier to find semantic correlations among elements that are already known rather than among elements in the adjacent possible space. In this way, there is a preference to head back to parts of the space that have been already visited. The two functions $f(N_\kappa, N_{\bar{\kappa}})$ and $g(N_\kappa, N_{\bar{\kappa}})$ instead, model the fact that the fraction of space accessible from highly visited elements is larger with respect to the same fraction from rarely visited ones. This is due to the fact that the exploration of the space is performed by a community and hence it is reasonable to assume that frequently visited elements are known by many different agents so that the shared knowledge of the space of novelties from those points is large.

Supposing that at time $t-1$, we have extracted the ball j with label κ and indicating the number of balls in the Adjacent Possible with the same label or triggered by j with m_j, it is possible to show that the number of distinct elements in $\mathcal{S}(t)$ at time t is governed by the equation:

$$\frac{dD_j(t)}{dt} = \frac{g(N_\kappa, N_{\bar{\kappa}})(m_j + \eta\nu(D_j(t) + N_0 - m_j))}{N_\kappa + \gamma f(N_\kappa, N_{\bar{\kappa}})N_{\bar{\kappa}} + g(N_\kappa, N_{\bar{\kappa}})(m_j + \eta(\nu D_j(t) + N_0 - m_j))}. \qquad (6)$$

In the limit $t \gg 1$, m_j is negligible. Hence, equation (6) becomes

$$\frac{dD_j(t)}{dt} = \frac{g(N_\kappa, N_{\bar{\kappa}})\eta\nu D_j(t)}{N_\kappa + \gamma f(N_\kappa, N_{\bar{\kappa}})N_{\bar{\kappa}} + g(N_\kappa, N_{\bar{\kappa}})\eta\nu D_j(t)}, \tag{7}$$

In order to model the dynamics of innovation processes we need $D(t)$ to follow the Heap's law and hence to grow sub-linearly in time with an exponent β.
It is possible to show that choosing $f = g = 1$ allows for an estimate of the bounds of β. With this choice equation 7) becomes,

$$\frac{dD(t)}{dt} = \frac{\eta\nu D(t)}{N_\kappa + \gamma N_{\bar{\kappa}} + \eta\nu D(t)}. \tag{8}$$

Note that since at each time step we add ρ balls in the urn, hence the total number of balls with a color which has already appeard in $\mathcal{S}(t-1)$ is $N_\kappa + N_{\bar{\kappa}} = \rho t + D$. Using the fact that $N_\kappa + \gamma N_{\bar{\kappa}} = (1-\gamma)N_\kappa + \gamma\rho t + \gamma D$, we find

$$\frac{dD(t)}{dt} = \frac{\eta\nu D(t)}{(1-\gamma)N_\kappa + \gamma\rho t + (\gamma + \eta\nu)D(t)}. \tag{9}$$

The total weight N_κ of the elements with label κ will be equal to the sum of the total number of elements already appeared in $\mathcal{S}$ with that label at time t, d_κ, plus the weight associated to the total number of times n_κ that the label appeared, ρn_κ. We can now release the assumption of being on a node with a particular label by summing over all the possible labels considering the probability $P(\kappa)$ of visiting the label κ:

$$\frac{dD(t)}{dt} = \sum_\kappa P(\kappa)\frac{\eta\nu D(t)}{(1-\gamma)(\rho n_\kappa + d_\kappa) + \gamma\rho t + (\gamma + \eta\nu)D(t)}, \tag{10}$$

Note that for every κ, $d_\kappa \leq \nu + 1$, since the maximum number of elements with the same label is $\nu + 1$, thus this term is negligible in the limit of large t. Moreover, we can shift from considering the probability of visiting κ, to the probability of visiting a generic label appeared n times:

$$\frac{dD(t)}{dt} = \sum_n P(n)\frac{\eta\nu D(t)}{(1-\gamma)\rho n + \gamma\rho t + (\gamma + \eta\nu)D(t)}, \tag{11}$$

We can then consider two limit cases in order to estimate the bounds of β. If the frequency-rank distribution is steep enough, we can consider just the terms with $n \sim t$ of the sum, so that

$$\frac{dD(t)}{dt} = \frac{\eta\nu D(t)}{\rho t + (\gamma + \eta\nu)D(t)} \tag{12}$$

gives an estimate $\beta = \min(1, \frac{\eta\nu}{\rho})$. In the opposite case, we must consider just the terms with $n \ll t$, so that

$$\frac{dD(t)}{dt} = \frac{\eta\nu D(t)}{\gamma\rho t + (\gamma + \eta\nu)D(t)} \tag{13}$$

gives an estimate $\beta = \min(1, \frac{\eta\nu}{\gamma\rho})$. Thus we have $\beta \in [\min(1, \frac{\eta\nu}{\rho}), \min(1, \frac{\eta\nu}{\gamma\rho})]$.
The choice $f = g = 1$ eliminates the modulation of the accessible space due to the presence of an exploring community of individuals. We can show that with a particular choice of the functions $f(N_\kappa, N_{\bar{\kappa}})$ and $g(N_\kappa, N_{\bar{\kappa}})$, it is possible to reshape equation (7) in (8) If we impose that the rhs of equation (7) is the same as the one in equation (8), we find that this is guaranteed if holds the relation:

$$g(N_\kappa, N_{\bar{\kappa}}) = \frac{N_\kappa + \gamma f(N_\kappa, N_{\bar{\kappa}})N_{\bar{\kappa}}}{N_\kappa + \gamma N_{\bar{\kappa}}}. \tag{14}$$

Note that as long as $f \in [0, 1]$, then also $g \in [0, 1]$. Moreover if f is an increasing function of N_κ then also g will be so. The choice of f is arbitrary and in the main text we have chosen

$$f(N_\kappa, N_{\bar{\kappa}}) = \frac{N_\kappa}{N_\kappa + \gamma N_{\bar{\kappa}}}, \tag{15}$$

but other choices are possible, like

$$f(N_\kappa, N_{\bar{\kappa}}) = \frac{N_\kappa}{N_\kappa + N_{\bar{\kappa}}}, \tag{16}$$

which is independent from γ. As far as the relation (14) holds equation (7) is equivalent to equation (8) and hence $\beta \in [\min(1, \frac{\eta\nu}{\rho}), \min(1, \frac{\eta\nu}{\gamma\rho})]$ is true also for the general model. This result is confirmed by the numerical simulations, as shown in Fig. G. Moreover, the relation between the Heap's law exponent (a-c panels) and the exponents of the tail of the Zipf's law (b-d panels) holds correctly as for the empirical datasets shown in Fig. A. We also checked

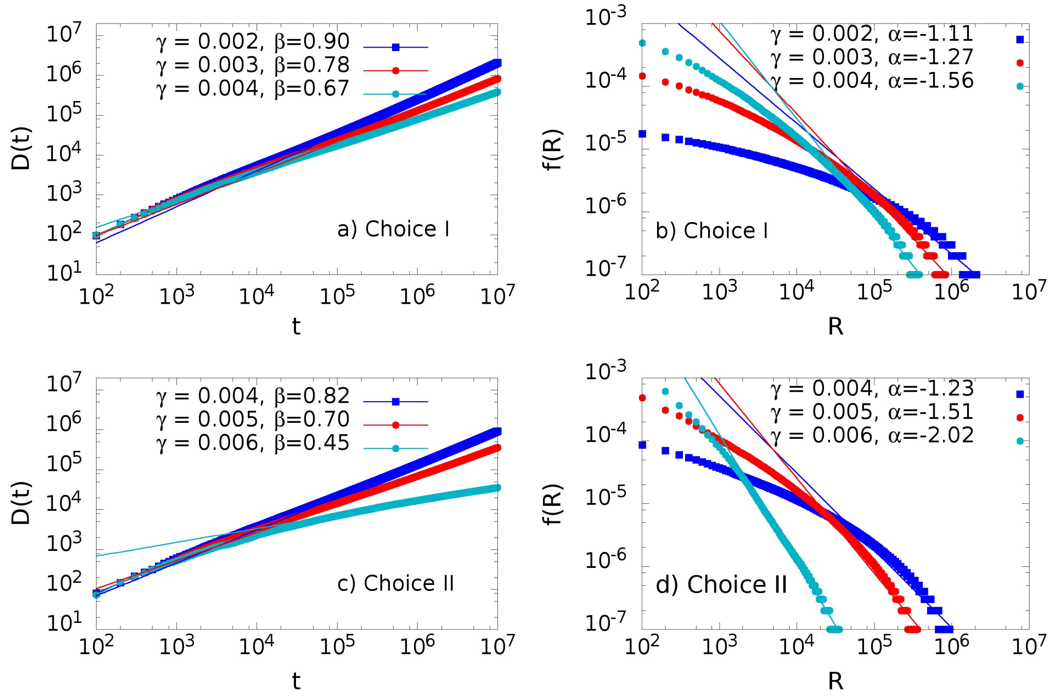

Figure G: **Heap's and Zipf's laws for the GUMT.** Left (a,c): Numerical results for the number of distinct elements as a function of the sequence length (Heaps' law). Right (b,d): Numerical results for the frequency rank distribution (Zipf's law). Top (a,b): results are reported for the choice I of the function f regulating the time dependent connectivity of the graph (refer to the main text) with $\rho = 1$, $\nu = 500$, $\eta = 0.001$ and $\gamma = 0.2, 0.3, 0.4$. Bottom (c,d): results are reported for the choice II of the function f with $\rho = 2$, $\nu = 15$, $\eta = 0.001$ and $\gamma = 0.004, 0.005, 0.006$.

that for some values of the parameter γ we are able to obtain a growth of $D(t)$ and values of δS compatible with the empirical findings. In Fig. H we show the behaviour of the exponent of the Heap's law β and the values of the semantic correlations metric δS as γ varies, comparing them with the values obtained with our datasets. In order to be consistent with Fig. 4 in the main text, we divided the datasets in Non-Textual and Textual ones. We find that the value of δS is almost stable and compatible with the datasets for a wide range of values of γ, on the other hand β is correctly reproduced for a smaller range of values. Finally, we can see that despite the same growth of $D(t)$ in the case $f = g = 1$ and in the general case, the behavior of the two models differs. Unlike the UMT model, the GUMT features two additional ingredients: an asymmetry in the semantic correlations between the already known elements and the adjacent possible ones, and a modulation of the semantic correlation parameters with the frequency of occurrence of a certain label.

Removing the first ingredient means choosing $\gamma = \eta$, leaving the modulation with the functions f and g. In Fig. I we show the comparison between the model with this choice and the UMT version. We see that despite some slight differences like an increase in the local entropy and in δS, the behaviour is qualitatively similar to what has been observed in section E. Observables as R and G in fact, indicate a small dominance of the firstly introduced elements

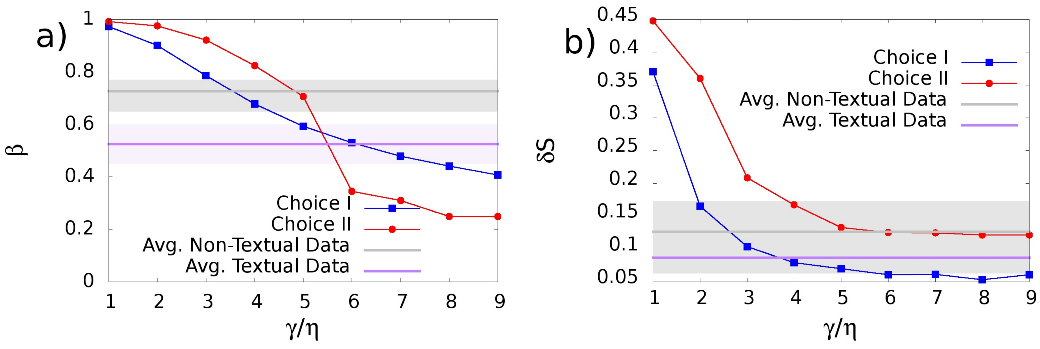

Figure H: **Heap's Exponent and Semantic Correlations** Exponent of the Heap's law β and δS as function of the ratio γ/η. The blue lines are the results for the choice I of the function f regulating the time dependent connectivity of the graph (refer to the main text) with $\rho = 1$, $\nu = 500$, $\eta = 0.001$, while the red lines are the results for the choice II of the function f with $\rho = 2$, $\nu = 15$, $\eta = 0.001$. The horizontal black line in each panel indicates the average value of the presented popularity metrics measured on Last.fm, Twitter and GitHub datasets, while the highlighted grey area is the range of such metrics limited by the maximum and minimum measured values. The horizontal purple line represents the same average for the Wikipedia and Gutenberg dataset, being the highlighted purple area the range defined by the waves of novelties metrics on these two datasets.

just in a region where the growth of $D(t)$ is linear, in contrast with the empirical findings. The removal of the second ingredient corresponds instead to choosing $f = g = 1$, leaving the asymmetry in the semantic correlations. Fig. J shows the comparison between the main-text model and the one with $f = g = 1$. In this case we can see that despite the model is still capable of showing some degree of variability in popularity and weakening in the dominance of first appeared elements, values really compatible with the data only emerge when the growth of $D(t)$ is almost linear, especially when considering the metrics R.

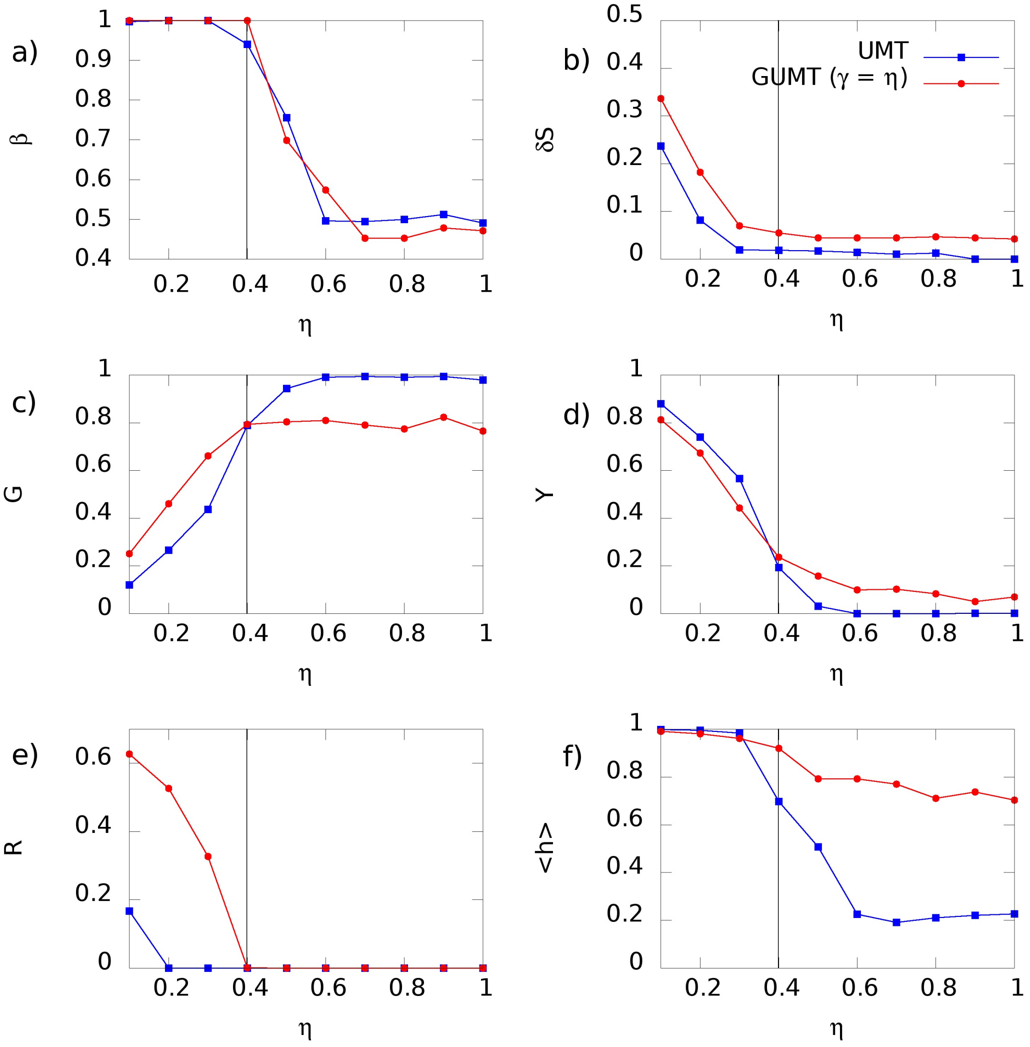

Figure I: **Comparison between the UMT and with the GUMT with** $\gamma = \eta$ Numerical Results for the model with $\gamma = \eta$ and $f(N_\kappa, N_{\bar{\kappa}}) = g(N_\kappa, N_{\bar{\kappa}}) = 1$ (red line, i.e. the model introduced in [7]) and with $\gamma = \eta$ and the choice I of $f(N_\kappa, N_{\bar{\kappa}})$ (blue line). Heap's Law growth exponent β (a), δS (b), G (c), Y (d), R (e), $\langle h \rangle$ (f) as functions of the semantic correlations parameter η. Black vertical lines indicates the largest value of η for which $\beta = 1$. All the simulations have been performed for 2.5×10^6 time steps. The values of the parameters correspond to the same choice of the blue curves in Fig. F.

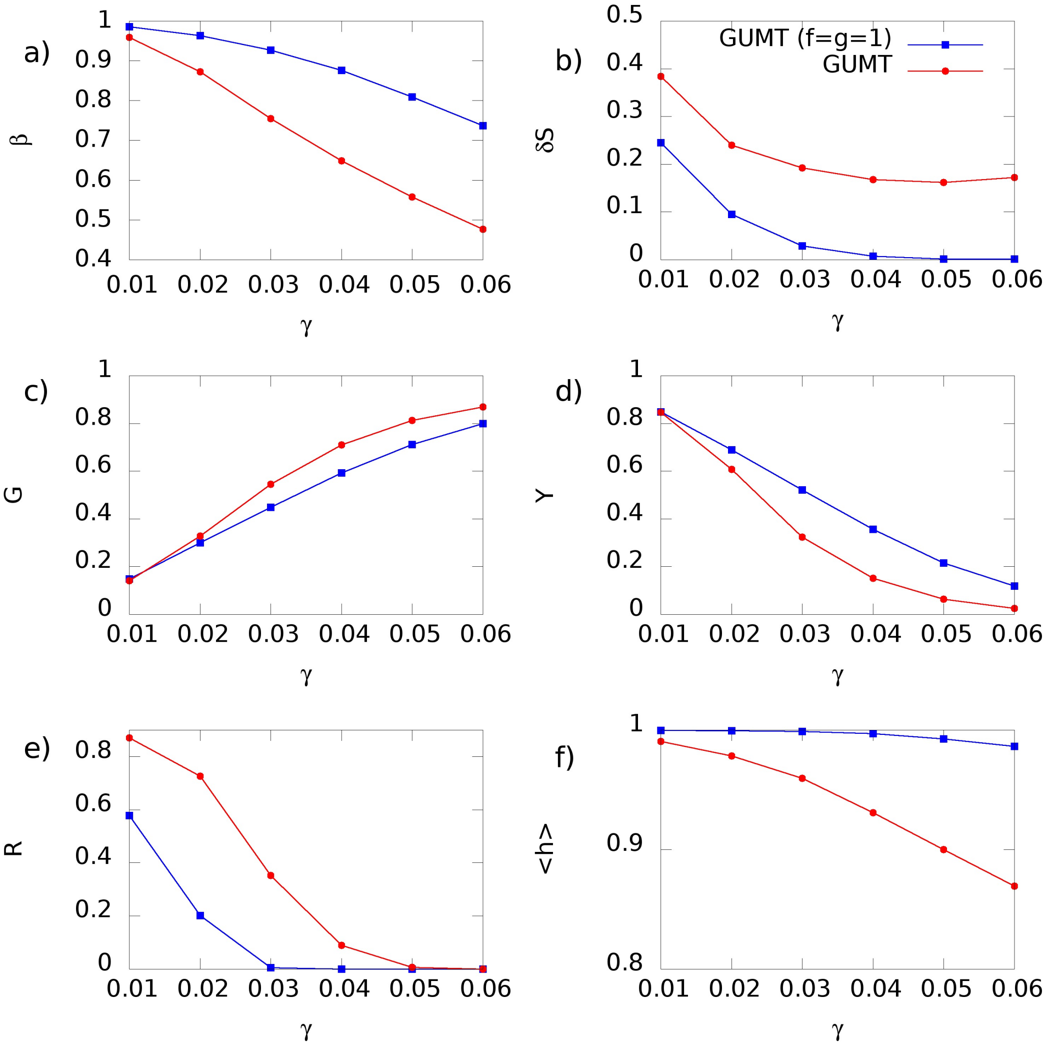

Figure J: **Comparison between the GUMT with $f = g = 1$ with and with GUMT in the general case.** Numerical Results for the model with $\gamma > \eta$ and $f(N_\kappa, N_{\bar{\kappa}}) = g(N_\kappa, N_{\bar{\kappa}}) = 1$ (red line) and with $\gamma > \eta$ and the choice II of the function f (blue line). Heap's Law growth exponent β (a), δS (b), G (c), Y (d), R (e), $\langle h \rangle$ (f) as functions of the semantic correlations parameter η. The simulations have been performed by choosing $\rho = 2$,$\nu = 100$ and $\eta = 0.001$.

G Graph Version of the GUMT

In [7] a mapping of the UMT modelling scheme has been introduced where the underlying space is modelled as a graph. In this version the expansion of the adjacent possible space corresponds to the expansion of an actual graph and the extraction of a ball from the urn corresponds to a visit of a specific node of the network. This modelling scheme resemble the one introduced in [11] in which different agents explores a network of related elements. However, in this case all the agents are considered merged into a single one, representing the whole community discovering the network. Here every time a new node appears in the graph new permanent links to other nodes are also drawn with a probability equal to η. We can of course imagine a graph-based version also for the GUMT model and this is what we present now with the crucial difference that links are not frozen forever, but redrawn instead at each time step.
In this scheme the urn is represented as a graph $\mathcal{G}$ with N_0 nodes divided in $\frac{N_0}{\nu+1}$ cliques with different labels assigned, being ν an integer number. We assign a weight $w_i = 1$ to each node at the beginning. Starting from a random position j in the graph the process is a random walk (RW) on this graph, which is also growing depending as the RW moves over it. Considering the sequence of visited nodes at time $t-1$, $\mathcal{S}(t-1)$, at each time step t:

(i) move the RW to a neighbour node i (including self-loops so the RW can stay on the present node) with a probability $p_i \propto w_i$;

(ii) reinforce the weight of the selected node so that $w_i \to w_i + \rho$, being ρ a positive real number and record it in $S(t)$;

(iii) if the node i was not already present in S (i.e., it is visited for the first time), add a new clique of $\nu+1$ nodes with the same labels and connect them to i. Each node of the clique is connected to all the other nodes already visited by the RW in the graph with a probability $\gamma f(N_\kappa, N_{\bar{\kappa}})$, where $f(N_\kappa, N_{\bar{\kappa}})$ is one of the functions introduced in section F. The nodes that have not already been visited are connected ti j with a probability $\eta g(N_\kappa, N_{\bar{\kappa}})$, where the relation (14) must hold between f and g. The parameters η and γ represent the semantic correlations between nodes with different labels, and $\gamma > \eta$ indicates that already visited nodes are more related than nodes that must be discovered.

In this picture the weight of each node represents the number of balls within the urn. For example, if before the weight of each ball whose colour appeared n_i times within $\mathcal{S}$ was $\gamma f(N_\kappa, N_{\bar{\kappa}})$ if they did not have the same label of the last extracted ball, now the weight of the node i will be $w_i = (\rho n_i + 1)$ (where in the total number of balls with the same color of i in the Urn would have been $\rho n_i + 1$ in the Urn Model case), but i will be reachable from j only with a probability $\gamma f(N_\kappa, N_{\bar{\kappa}})$. Figure K shows a pictorial representation of the network model.
We have not found significant numerical differences between the Urn and the Graph versions as can be seen in figure L. Both the growth of $D(t)$ and the frequency-rank plots are usually strikingly similar. In the presented case we have: a Gini-like coefficient of 0.59 for the Graph version and 0.55 for the Urn version; a Youth coefficient of 0.34 for the Graph version and 0.36 for the Urn one; a value of Recentess of 0.38 for the Graph version and 0.44 for the Urn one; and finally a local entropy of 0.95 for the Graph version and 0.96 for the Urn one.
Finally, Fig. M shows the results of the same numerical simulations of Fig. 4 in the main-text but for the Graph-based model. Such results are again qualitatively consistent with the ones found in the urn case.

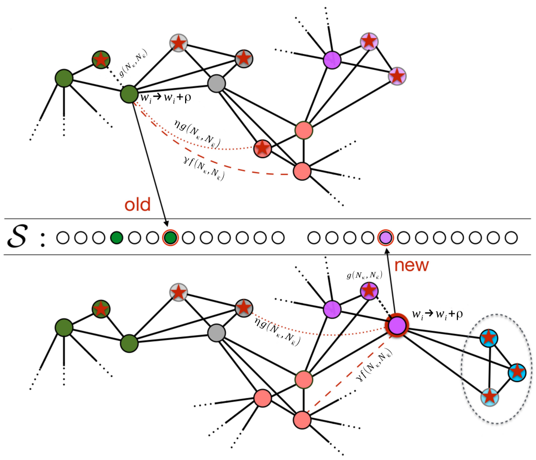

Figure K: **Pictorial Representation of the Graph Model.** In this model, the dynamics is a Random Walker on a growing graph. Whenever an element is visited its weight is increased by a value ρ. If an element is visited for the first time, a clique of $\nu+1$ elements connected to it and sharing the same label is introduced in the network. Supposing that the last visited node has a certain label (indicate with the color green in the picture), then: the nodes in the adjacent possible (denoted by stars in the picture) are connected to the current one with probability equal to $g(N_\kappa, N_{\bar{\kappa}})$ if they share the same label as the last visited node and with $\eta g(N_\kappa, N_{\bar{\kappa}})$ otherwise; the already visited nodes are connected with probability 1 if they share the same label as the last visited node and with $\gamma f(N_\kappa, N_{\bar{\kappa}})$ otherwise. Note that $\gamma > \eta$ and N_k depends on the number of times the clique has been visited.

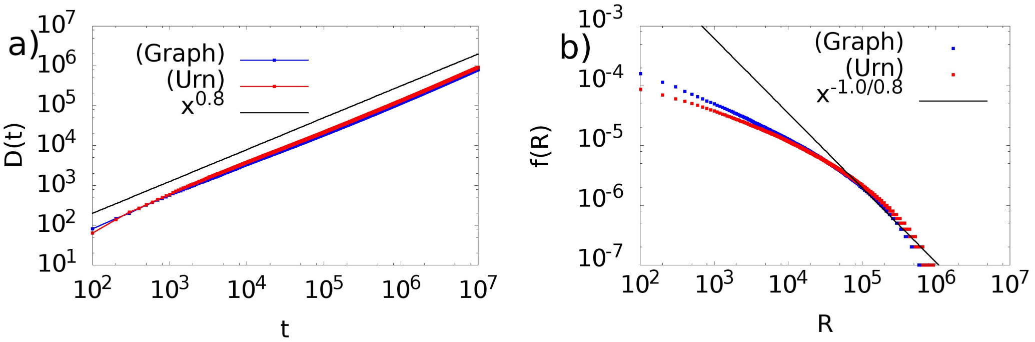

Figure L: **Comparison between the Graph Model and the Urn Model.** (a) Growth of the different number of elements and (b) Freque ncy-Rank plot for the model introduced in the Network and the Polya Urn version of the same model. Simulations correspond to the choice II of the function f with $\rho = 2$, $\nu = 15$, $\eta = 0.001$ and $\gamma = 0.005$.

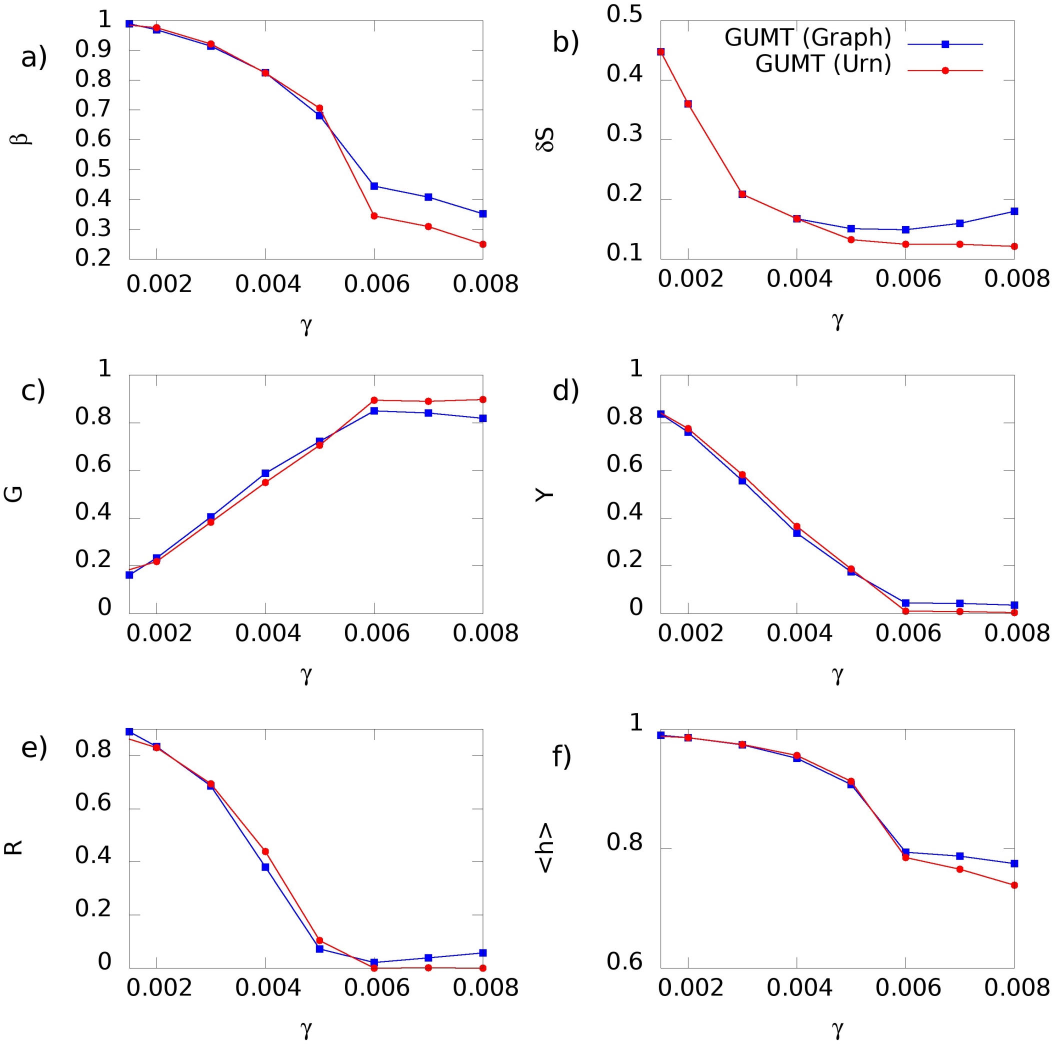

Figure M: **Numerical Results for the Graph Model in the same cases as Fig. 4 of the Main Text** Numerical results for: Heap's law exponent β (a), δS (b), G (c), Y (d), R (e), $\langle h \rangle$ (f), for different values of the parameter γ and the two choices of the function f (blue line for the choice I and red line for the choice II). The other parameters of the model are kept fixed to the values: $\rho = 2$, $\nu = 15$, $\eta = 0.001$.

H Number of Users and Appearance Number

For the three datasets where it is possible to define a user, i.e., Last.fm, Twitter and Github, we report in Fig. N a clear correlation between the number of different users that contributed to/have interacted with a certain element and the number of times such element appeared. This correlation strengthen the hypothesis that the number of appearances of a given element can be used as a proxy for the number of users who knew a given element. We used this fact in order in our modelling scheme GUMT to modulate the semantic correlation as an effect of the presence of multiple agents in the system.

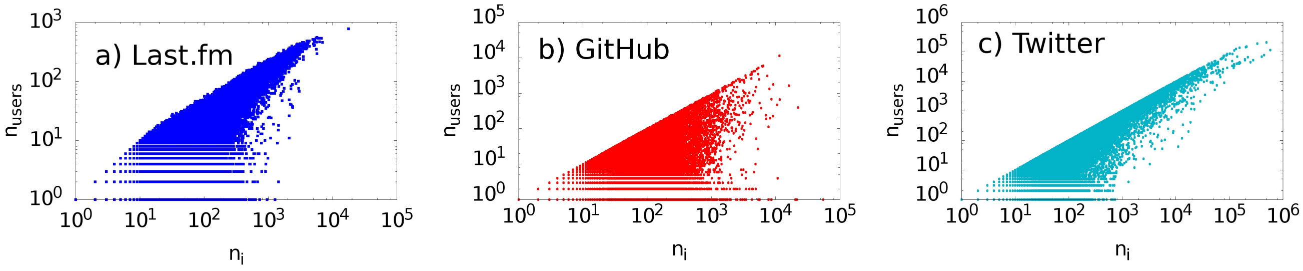

Figure N: **Correlations between the appearance number of an element and the number of users that have interacted with it.** Plot of the number of times an element appeared (x-axis) and the number of users that have interacted with it (y-axis) for the Last.fm dataset (a), the GitHub dataset (b) and the Twitter dataset (c). The Pearson's correlation coefficient measured in each case are respectively $\rho = 0.89$ (a), $\rho = 0.42$ (b) and $\rho = 0.84$ with a p-value considerably smaller than 0.01.

References

[1] Last.fm;. Accessed: May 2012. URL http:/last.fm Dump from Music Recommendation Datasets for Research: URL http://www.dtic.upf.edu/~ocelma/MusicRecommendationDataset/.

[2] Weng L, Menczer F. Topicality and Impact in Social Media: Diverse Messages, Focused Messengers. PLoS ONE. 2015;10(2):e0118410.

[3] GitHub;. Accessed: May 2015. URL https:/github.com Dump from GitHub Archive: URL https://www.githubarchive.org/.

[4] Wikipedia;. Accessed: April 2015. URL http://www.wikipedia.org/ English Dump from: URL http://dumps.wikipedia.org/enwiki/20120307/.

[5] Functionwords;. Accessed: April 2015. List of 200 function words from: URL http://ebooks.cambridge.org/chapter.jsf?bid=CBO9780511605437&cid=CBO9780511605437A021.

[6] Hart M. Gutenberg;. Accessed on February 2007. URL http://www.gutenberg.org/.

[7] Tria F, Loreto V, Servedio VDP, Strogatz SH. The dynamics of correlated novelties. Scientific Reports. 2014;4.

[8] Monechi B. All datasets dump;. Diofa. URL http://kreyon.net/waves-of-novelties/.

[9] Heaps HS. Information retrieval: Computational and theoretical aspects. Academic Press, Inc.; 1978.

[10] Blei DM, Ng AY, Jordan MI. Latent dirichlet allocation. Journal of machine Learning research. 2003;3(Jan):993–1022.

[11] Saracco F, Di Clemente R, Gabrielli A, Pietronero L. From innovation to diversification: a simple competitive model. PloS ONE. 2015;10(11):e0140420.
